# High-throughput computational stacking reveals emergent properties in natural van der Waals bilayers

**DOI:** 10.1038/s41467-024-45003-w

**Published:** 2024-01-31

**Authors:** Sahar Pakdel, Asbjørn Rasmussen, Alireza Taghizadeh, Mads Kruse, Thomas Olsen, Kristian S. Thygesen

**Affiliations:** CAMD, Computational Atomic-Scale Materials Design, Department of Physics, Technical University of Denmark, 2800 Kongens Lyngby, Denmark

**Keywords:** Two-dimensional materials, Electronic properties and materials, Magnetic properties and materials, Ferroelectrics and multiferroics, Computational methods

## Abstract

Stacking of two-dimensional (2D) materials has emerged as a facile strategy for realising exotic quantum states of matter and engineering electronic properties. Yet, developments beyond the proof-of-principle level are impeded by the vast size of the configuration space defined by layer combinations and stacking orders. Here we employ a density functional theory (DFT) workflow to calculate interlayer binding energies of 8451 homobilayers created by stacking 1052 different monolayers in various configurations. Analysis of the stacking orders in 247 experimentally known van der Waals crystals is used to validate the workflow and determine the criteria for realisable bilayers. For the 2586 most stable bilayer systems, we calculate a range of electronic, magnetic, and vibrational properties, and explore general trends and anomalies. We identify an abundance of bistable bilayers with stacking order-dependent magnetic or electrical polarisation states making them candidates for slidetronics applications.

## Abstract (web summary)

2D bilayers have recently attracted significant attention due to fundamental properties like interlayer excitons and interfacial ferroelectricity. Here, the authors report a density functional theory approach to identify 2586 stable homobilayer systems and calculate their stacking-dependent electronic, magnetic and vibrational properties.

## Introduction

The field of 2D materials has evolved at a tremendous pace over the past decade and is currently impacting many areas of contemporary physics including spintronics^1,2^, valleytronics^3^, polaritonics^4^, unconventional superconductivity^5^, multiferroics^6^, and quantum light sources^7^. While numerous 2D monolayers have been extensively scrutinised in experiments and their properties systematically organised in computational databases^8,9^, studies of 2D multilayer structures have been much more sporadic.

The unit cell commensurate homobilayers (from hereon referred to as natural bilayers or simply bilayers) represent a well-defined and highly interesting class of 2D multilayer materials. Despite sharing the same Bravais lattice as the monolayer, their point group symmetry can differ depending on the stacking order. Such qualitative differences can influence physical properties profoundly with direct consequences for the material’s utilisation potential^10–12^. For example, non-volatile ferroelectric memories^13^, the valley Hall effect^14^, and spontaneous valley polarisation^15^, require materials with broken inversion symmetry. This may be achieved in a homobilayer even if the monolayer is centrosymmetric. Furthermore, due to the presence of the van der Waals (vdW) gap, the properties of bilayers can be tuned more effectively as exemplified by the giant Stark effect of interlayer excitons in bilayer MoS_2_^16,17^, switching of magnetic states in bilayer CrI_3_ by either electrical gating^18,19^ or pressure^20^, and coupled ferroelectricity-superconductivity in bilayer MoTe_2_^21^.

It has recently been proposed that the layer degree of freedom in vdW bilayers could form the basis for a distinct type of 2D interfacial ferroelectrics^22–24^. Subsequently, interfacial ferroelectricity has been demonstrated in bilayers of hexagonal boron nitride^25,26^ and transition metal dichalcogenides (TMDs)^27–29^. In these experiments, two stacking configurations with different out-of-plane polarisations are electrically switched via in-plane sliding of the layers. Beyond out-of-plane ferroelectricity, one might envision slide-induced switching of other physical quantities such as in-plane polarisation, conductivity, magnetism, or band topology^30,31^. Further progress in the emerging field of slidetronics^32–34^ calls for the identification of specific bilayer systems with (meta)stable stacking configurations separated by low switching barriers.

While the production of vdW heterostructures and twisted bilayers is a complex and time-consuming process with low yield and high sample-to-sample variability, consistent high-quality homobilayers may be exfoliated from naturally occurring bulk samples or grown bottom-up by scalable chemical methods^35,36^. Despite of this clear advantage and the exciting prospects described above, the homobilayers remain largely unexplored compared to their simpler monolayer constituents.

Here, we employ a first-principles high-throughput workflow to systematically construct and explore all homobilayers that can be formed from 1052 monolayers. Our bottom-up stacking workflow yields a total of 2586 bilayers that we predict to be stable and experimentally realisable based on an extensive analysis of the stacking orders found in 247 known vdW bulk compounds. We analyse the emergent properties of the bilayers (relative to their monolayers) and discover an abundance of systems possessing stacking order-dependent electronic, magnetic, or ferroelectric properties - some of which are switchable via layer sliding. The full library of homobilayers will be available as an open database fully integrated with the C2DB monolayer database, making a unique digital platform to support and accelerate 2D materials science.

## Results

### Stacking workflow

**Fig. 1:**
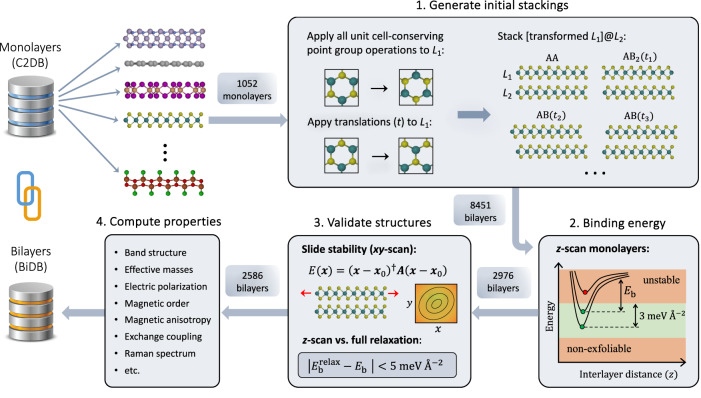
Stacking workflow. A total of 1052 stable monolayers are imported from the Computational 2D Materials Database (C2DB) with the condition that the number of atoms per unit cell (*N*_atoms_) does not exceed 10. Starting from the AA stacked bilayer, various stacking configurations are generated by applying all unit cell-preserving point group operations to layer 1 (*L*_1_) while keeping layer 2 (*L*_2_) fixed. For each of these configurations, ${N}_{{{{{{{{\rm{atoms}}}}}}}}}^{2}$ structures are generated by translating layer 1 by a vector *t*_*ij*_ given by the difference between the lateral positions of atoms *i* and *j* in the monolayer unit cell. Duplicate bilayers are subsequently removed. The interlayer binding energy, *E*_b_, of the 8451 unique bilayers is then obtained by scanning the PBE-D3 energy of the frozen monolayers as a function of the interlayer distance (*z*-scan approach). Bilayers with *E*_b_ within 3 meV/Å^2^ of the most stable stacking are considered thermodynamically stable. The 2976 thermodynamically stable bilayers are then validated by checking their stability against lateral sliding. This is done by verifying that the energy as a function of the lateral displacement (**x** − **x**_0_) of the frozen layers, is a quadratic function with positive definite Hessian (**A**). The reliability of the *z*-scan approach is also verified by ensuring that the binding energy resulting from the *z*-scan approach (*E*_b_) does not deviate from the binding energy obtained from a full relaxation of the bilayer (${E}_{{{{{{{{\rm{b}}}}}}}}}^{{{{{{{{\rm{relax}}}}}}}}}$) by more than 5 meV/Å^2^. The final 2586 bilayers are run through the property workflow.

Our stacking workflow starts by extracting 1052 stable monolayers with up to 10 atoms/unit cell from the C2DB^8,37^, and arranging them in a total of 8451 unique stacking configurations (see Fig. 1 and Methods). The interlayer distance and binding energy, *E*_b_, is determined for each bilayer by scanning the total energy as a function of the interlayer separation (“*z*-scan” approach). The vast majority of the constructed bilayers have *E*_b_ below 50 meV/Å^2^ indicating interlayer bonds of purely vdW character. This is a result of the stringent stability criteria we used to select the monolayers. Indeed, a monolayer with good thermodynamic stability, i.e., low formation energy, is unlikely to have dangling bonds and thus unlikely to form strong interlayer bonds. A bilayer with interlayer binding energy *E*_b_ is considered thermodynamically stable if $\Delta {E}_{{{{{{{{\rm{b}}}}}}}},\max }\equiv {E}_{{{{{{{{\rm{b}}}}}}}},\max }-{E}_{{{{{{{{\rm{b}}}}}}}}} \, < \, 3$ meV/Å^2^, where ${E}_{{{{{{{{\rm{b}}}}}}}},\max }$ is the maximum binding energy among all the considered stacking configurations. We shall return to this criterion below. Further details, motivation, and justification for the *z*-scan approach, including comparison to the fully relaxed structures, can be found in Methods and the Supplementary information.

Next, the slide stability is checked for all thermodynamically stable bilayers by fitting the local potential energy surface to a second-order polynomial (see Supplementary section C for details). Unstable configurations are pushed along a negative gradient (or curvature for saddle points), and a constrained energy minimisation is performed keeping the monolayers frozen. The procedure is repeated until a slide stable configuration is obtained. After removing duplicate structures, we obtain 2586 unique thermodynamically and slide stable bilayers, from hereon referred to as *stable bilayers*.

**Fig. 2:**
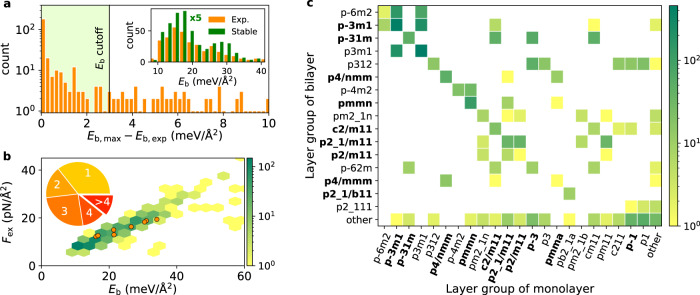
Bilayer stability, exfoliation force, and crystal symmetry. **a** Histogram showing the difference between the interlayer binding energy of bilayers in an experimentally observed stacking configuration $({E}_{{{{{{{{\rm{b}}}}}}}},\exp })$ and the most stable stacking configuration predicted by the stacking workflow (${E}_{{{{{{{{\rm{b}}}}}}}},\max }$). Stackings with a binding energy within the cutoff of 3 meV/Å^2^ from ${E}_{{{{{{{{\rm{b}}}}}}}},\max }$ (green shaded region) are considered thermodynamically stable in this work (note the logarithmic scale). The inset shows the distribution of the binding energies calculated for the experimentally observed stackings (orange) and all the predicted stable bilayers (green). The green distribution has been reduced by a factor 5. **b** Exfoliation force (*F*_ex_) versus interlayer binding energy: Exfoliation forces are shown for 20% of the stable bilayers for which we specifically calculated more data points on the *E*_b_(*z*) curve. The exfoliation force is the minimum force per area required to pull the bilayer apart by vertical lift-off. A number of known exfoliable 2D materials are indicated by orange dots (Graphene, Phosphorene, BN, MoS_2_, NbSe_2_, PtSe_2_, WTe_2_, HfSe_2_). The inset shows the number of stable bilayers obtained per monolayer. **c** Matrix representation of the layer group of stable bilayers versus the layer group of the constituent monolayer. The layer groups with inversion symmetry are marked in bold font. The colour bars in panels **b** and **c** represent the number of bilayers.

We now address the question: when can a stacking configuration be expected to be experimentally realisable? Clearly, the energy of such configurations should not be too high above that of the most stable stacking configuration. The binding energy of the latter is denoted ${E}_{{{{{{{{\rm{b}}}}}}}},\max }$. To determine a reasonable bound on $\Delta {E}_{{{{{{{{\rm{b}}}}}}}},\max }$, we analyse its value for stacking orders observed in naturally occurring vdW crystals. Figure 2a shows the distribution of ${E}_{{{{{{{{\rm{b}}}}}}}},\max }-{E}_{{{{{{{{\rm{b}}}}}}}},\exp }$, i.e., the binding energy of bilayers in the experimentally observed stacking relative to the most stable stacking found by our workflow (note the logarithmic scale). The analysis includes 247 experimental bulk structures and 226 monolayers (21 monolayers appear twice in different stacking patterns). From the 247 bulk structures, we extract 314 bilayers. Note that bulk crystals with more than two monolayers per unit cell can result in more than one unique bilayer.

For 73% of the 226 monolayers that make up the set of experimentally known bulk crystals, the most stable bilayer found by the stacking workflow, coincides with an experimentally observed stacking (leftmost bar in Fig. 2). However, there are also cases where an experimentally observed stacking has an energy above the predicted most stable stacking. This could be due to several reasons, e.g., differences in interlayer interactions for bulk and bilayer (in Supplementary Fig. 1 we show that this effect is very small), limited accuracy of the calculations, finite temperature effects not accounted for in the calculations, or the occurrence of meta-stable stacking orders in the natural bulk crystals. To encompass such effects while avoiding to include too many unphysical/unstable structures, we require that $\Delta {E}_{{{{{{{{\rm{b}}}}}}}},\max } \, < \, 3$ meV/Å^2^. Out of the 314 bilayer stackings found in the experimental bulk crystals, 74% satisfy this condition.

The distributions of *E*_b_ for all the stable bilayers and the bilayers in experimentally observed stacking configurations are shown in the inset of Fig. 2a. The similarity of the two distributions supports our approach for selecting stable, experimentally realisable bilayers.

The *z*-scan approach allows us to fit *E*_b_(*z*) by an analytical Buckingham potential for the vdW interaction between two 2D planes, and thereby determine the exfoliation force, *F*_ex_ (see Methods and Supplementary section B). Figure 2b shows, not unexpectedly, that *F*_ex_ is correlated with *E*_b_. However, there are also deviations from a linear relationship implying that *E*_b_ is not always an accurate descriptor for exfoliability.

The pie chart in the inset of Fig. 2b shows the number of stable stackings found per monolayer. Interestingly, for about 2/3 of the monolayers, our workflow predicts the existence of multiple stable stacking configurations. Such bilayers could form the basis for novel switchable systems as will be discussed later.

As mentioned, the crystal symmetry is of key importance to many applications of 2D materials. In Fig. 2c we show the layer group type of the monolayer versus the layer group type of the bilayer. It can be seen that the crystal symmetry often changes upon stacking. In particular, the breaking/emergence of inversion symmetry (indicated by bold font) occurs frequently when monolayers are stacked and could lead to qualitatively different physical properties.

### Vibrational properties

Raman spectroscopy is one of the most important and widespread techniques for characterising 2D materials. In first-order Raman spectroscopy, the Γ-point phonons are probed via inelastic light scattering yielding detailed structural and electronic information from nondestructive measurements. While most Raman studies of layered vdW materials have focused on high-frequency intralayer phonons, low-frequency Raman spectroscopy is emerging as a means to probe interlayer couplings with higher precision^38^. To support these developments we systematically explore the sensitivity of Raman spectroscopy to interlayer coupling by calculating the full Raman tensor of 481 non-magnetic monolayers (with up to 8 atoms per monolayer cell) and their 1244 stable bilayers. The computational methodology is described in Methods and extensive benchmarks against experiments are presented in Supplementary section F.

**Fig. 3:**
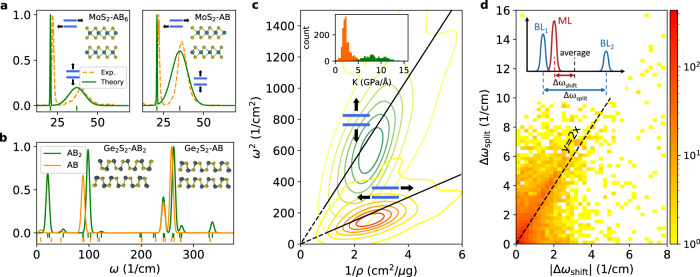
Raman spectra and statistics for interlayer and intralayer vibrational modes. **a** Low-frequency part of the Raman spectrum of bilayer MoS_2_ in the AB_6_ and AB stacking configurations, respectively. Side views of the atomic structure of the bilayers are shown as insets. The calculated spectra (full lines) are compared to the experimental spectra of ref. ^60^ (dashed lines). The broadening of the peaks in the theoretical spectra has been fitted to match the experiments. The in-plane shear and the out-of-plane breathing phonon modes are shown next to the respective Raman peaks. **b** Calculated Raman spectra of bilayer Ge_2_S_2_ in the two stacking configurations shown as insets. It can be seen that both the low and high-frequency parts of the Raman spectrum are sensitive to the stacking order. **c** Distributions of shear and breathing interlayer modes (illustrated by the blue bars and black arrows) as a function of the square of the phonon frequency, *ω*^2^, and inverse layer mass density, *ρ*^−1^. Only bilayers for which the two types of modes do not couple are included. The black lines have slopes 〈*K*_⊥_〉 = 9.42 and 〈*K*_∥_〉 = 2.64, corresponding to the mean value of the effective spring constants for the two distributions (shown in the inset). **d** Change in the monolayer optical phonon frequencies when stacked into bilayers. The plot shows the splitting (Δ*ω*_split_) versus the shift (Δ*ω*_shift_) of the monolayer phonon frequencies (see inset). The in-phase vibrations (BL_1_) are essentially unperturbed while the out-of-phase vibrations (BL_2_) are blue-shifted. The colour bar represents the number of bilayers on a logarithmic scale. The data points tend to cluster around the black dashed line corresponding to Δ*ω*_split_ = 2Δ_shift_ (see main text).

Any vdW bilayer supports three low-frequency interlayer modes: Two in-plane shear modes (degenerate for isotropic materials) and one out-of-plane breathing mode. As an example, Fig. 3a shows the calculated and experimental low-frequency Raman spectrum of bilayer MoS_2_ in AB_2_ (2H) and the AB (3R) stacking configurations (our bilayer notation is explained in Methods). Both the experimental and theoretical spectra show a significant difference in the relative peak amplitude in the two stacking configurations as well as a shift in frequency of the breathing mode of 2.0 cm^−1^ (calculation) and 3.5 cm^−1^ (experiment). Figure 3b compares the Raman spectra of bilayer Ge_2_S_2_ in two stacking configurations. Again, the low-frequency modes show significant frequency shifts, but even more drastic changes occur for the peak intensities due to different crystal symmetries. The high-frequency spectrum also shows differences, although to a lesser extent. These examples clearly illustrate the sensitivity of the interlayer modes to the interfacial vdW interactions and the potential of low-frequency Raman spectroscopy for identifying stacking orders.

Within the harmonic approximation, the frequency of an interlayer vibration fulfils *ω*^2^ = *K*/*ρ*, where *ρ* is the monolayer mass density and *K* is the effective spring constant quantifying the strength of the interfacial vdW interaction. Figure 3c shows the distributions of shear modes (orange) and breathing modes (green) of all bilayers as a function of *ω*^2^ and *ρ*^−1^. Lines with slopes equal to the mean of the two *K*-distributions (see inset) are shown. The shear modes have strikingly similar spring constants while a significantly larger spread is found for the breathing modes. The shown distributions include all bilayers where the two types of interlayer modes decouple. For the remaining ca. 250 bilayers, the shear and breathing modes mix revealing an intrinsic mechanical coupling between out-of-plane uniaxial strain and interlayer shear.

We now turn to the intralayer phonons. Each intralayer phonon of the monolayer will give rise to two phonons in the bilayer. Due to the interfacial vdW interactions, the phonons of the bilayer will split (Davydov splitting) and shift relative to the monolayer phonons, see inset of Fig. 3d. By projecting the phonon modes of the bilayer onto the modes of the isolated monolayers, we are able to track the phonon hybridisation and unambiguously determine the frequency split and shift for each individual mode. The enhanced intensity of the data points along the line Δ*ω*_split_ = 2Δ_shift_ (note the log scale) shows that in many cases the softening of the in-phase vibrations is weaker than the hardening of the out-of-phase vibrations. The extensive data set of Raman tensors provided in this work will advance the understanding and interpretation of vibrational spectroscopy of 2D materials.

### Electronic properties

The idea of modifying electronic band gaps via layer stacking has been paradigmatic in the field of 2D materials. Well-known examples include the indirect to direct band gap transition in group-VI TMDs^39^ and the metal-semiconductor transition in PtSe_2_^40,41^. In general, a qualitative change in the gap type has dramatic consequences for the material’s properties and possible applications. To systematically explore the opportunities and limitations of band gap stacking engineering, we have calculated the electronic band structure including the spin-orbit coupling for all bilayers (only the stable ones are included in the figure). Note that unstable stacking configurations are relevant as reference structures when building models for twisted bilayers.

One mechanism affecting the band gap upon stacking is interlayer hybridisation. This effect is controlled by the overlap of wave functions across the vdW gap and as such it is sensitive to both the interlayer distance and lateral stacking configuration. In addition to interlayer hybridisation, the band gap can be affected by the presence of an out-of-plane polarisation in polar bilayers. The latter effect can be quantified by the potential step across the bilayer created by the polarisation, Φ_P_.

**Fig. 4:**
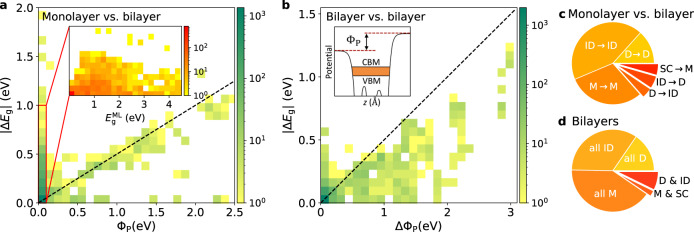
Trends in band gaps and electronic types. **a** Change in electronic band gap from monolayer to bilayer as a function of the out-of-plane polarisation potential across the bilayer, Φ_P_ (see inset of panel **b**, where CBM (VBM) stands for conduction band minimum (valence band maximum)). Only stable bilayers are shown in the plot. The dashed line indicates the situation where the bilayer gap is exactly 0.5Φ_P_ smaller than the monolayer gap. For the bilayers with zero out-of-plane polarisation, the gap change is shown in the inset as a function of the monolayer band gap. The colour bars in panels **a** and **b** show the number of bilayers on a logarithmic scale. **b** The change in band gap between different stable stackings of the same monolayer plotted as a function of the difference in the out-of-plane dipole of the two bilayers, ΔΦ_P_. **c** The distribution of bilayers according to the change in electronic type from monolayer to bilayer (M: Metal, SC: Semiconductor, D: Direct band gap, ID: Indirect band gap). There are 349 bilayers with an electronic type different from their monolayer. **d** The distribution of monolayers according to the electronic types of all its stable bilayers. There are 91 monolayers with stacking-dependent electronic types (D & ID or M & SC).

Figure 4a shows the change in band gap, Δ*E*_g_, from monolayer to bilayer as a function of Φ_P_. Except for 10 bilayers, the gap is always reduced upon stacking. The common feature of the 10 anomalous monolayers is the presence of hydrogen atoms on the surface, which upon stacking interpenetrates leading to a strong and complex hybridisation. Bilayers with large Φ_P_ consist of monolayers with broken mirror symmetry (Janus monolayers) stacked with aligned dipoles. Except for the metals, characterised by Δ*E*_g_ = 0, these bilayers undergo a band gap reduction of ≈ 0.5Φ_P_. This occurs due to the offset of the bands in the two layers produced by the polarisation potential. For bilayers with Φ_P_ = 0, the gap change is solely driven by hybridisation. For this subset, the gap change shows a decreasing trend as a function of the monolayer gap (see inset). This can be understood as a reduced strength of valence-conduction band hybridisation across the vdW gap. The change in electronic type upon stacking is shown in panel (c). A total of 349 bilayers undergo either a change in band gap type or a semiconductor-metal transition upon stacking. In particular, we find 126 bilayers with an emergent direct gap (composed of indirect gap monolayers) of interest for applications in nanophotonics and opto-electronics.

Figure 4b shows the difference in band gap between two stable stacking configurations of the same monolayer as a function of the difference in polarisation potential, ΔΦ_P_. Despite the weakness of the vdW interaction, stacking-dependent gap changes of up to 0.5 eV can arise purely from interlayer hybridisation (ΔΦ_P_ = 0). For bilayer pairs with finite dipole differences (ΔΦ_P_ > 0), gap changes of up to 1 eV occur when Janus monolayers, such as MoSSe, are stacked with parallel and anti-parallel dipole orientations, respectively. As can be seen in panel (d), most bilayers exhibit the same electronic type in all (stable) stacking configurations. However, 91 monolayers (~10%) support stacking configurations with different, and potentially switchable, electronic types.

### Magnetic properties

Magnetically ordered bilayers constitute a highly attractive platform for designing versatile building blocks in next-generation spintronic devices, such as spin filter magnetic tunnel junctions^42^ and spin tunnel field effect transistors^43^. The interfacial vdW coupling allows for possible ferromagnetic (FM) or antiferromagnetic (AFM) orders. We write the energy difference between the two magnetic states as

1 $${E}_{{{{{{{{\rm{FM}}}}}}}}}-{E}_{{{{{{{{\rm{AFM}}}}}}}}}=2{N}_{{{{{{{{\rm{a}}}}}}}}}\,J{S}^{2},$$

which defines the effective interlayer exchange coupling *J*. Here *S* is the average spin per magnetic atom and *N*_a_ is the number of magnetic atoms in the monolayer unit cell, see Supplementary section H for details. Accurate determination of *J* from first principles is challenging as it depends sensitively on the stacking configuration, temperature, and the model used for exchange-correlation effects. In Supplementary Table 1 we show that our calculations correctly predict the experimentally observed magnetic order (FM or AFM) in seven out of nine magnetic homobilayers.

**Fig. 5:**
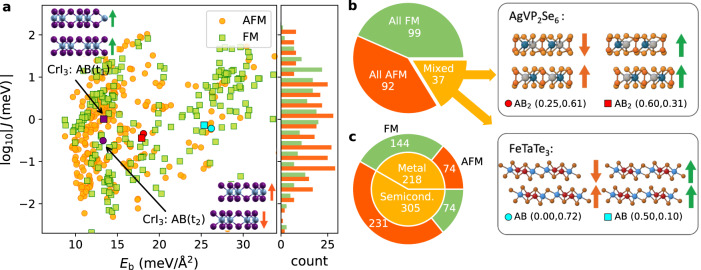
Magnetic properties. **a** Interlayer exchange coupling versus interlayer binding energy for the most stable, magnetic bilayer stackings. The orange (green) symbols represent bilayers with FM (AFM) order being lowest in energy. The crystal structure of the two experimentally observed stacking configurations (also predicted as the two most stable) of bilayer CrI_3_ are shown. The data points corresponding to the bilayer structures in panel **b** are highlighted with red and cyan symbols. **b** Fraction of magnetic monolayers for which the magnetic order of all stable stackings is either FM (green), AFM (orange), or mixed (yellow). From the latter group, AgVP_2_Se_6_ and FeTaTe_3_ are highlighted as examples of bilayers that may be switched between FM and AFM magnetic configurations by sliding. **c** Distribution of all stable bilayer stackings according to magnetic order (FM/AFM) and electronic type (metal/semiconductor).

Figure 5a shows *J* versus *E*_b_ for 595 stable magnetic bilayers. The lack of any clear correlation between the two quantities suggests that they are governed by different physical mechanisms. Indeed, *E*_b_ is governed by vdW interactions, which is a non-local correlation effect, while *J* is an exchange effect, which depends on the spatial overlap of the orbitals carrying the magnetic moments in the two layers.

We find 37 monolayers with stable bilayers exhibiting FM or AFM order depending on the stacking configurations (Fig. 5b). One of these materials is the well-known CrI_3_. Among the remaining bilayers, we highlight AgVP_2_Se_6_ and FeTaSe_3_. Both materials are experimentally known in bulk form and the relatively small *E*_b_ indicates that they should be exfoliable. According to the C2DB, monolayer FeTaTe_3_ is metallic with in-plane magnetic easy axis, while monolayer AgVP_2_Se_6_ is a semiconductor with FM order, out-of plane easy axis, and in-plane ferroelectric order^44^. For both materials, the interlayer FM and AFM stacking configurations are related by a pure translation implying that the magnetic state could be switched by sliding.

Interestingly, monolayers with broken mirror symmetry also form bilayers with stacking-dependent magnetic order. For example, bilayers of the Janus monolayer VTeS are metallic and the built-in dipole leads to charge transfer, which affects the magnetic properties of the two constituent monolayers. In particular, one stacking yields a ferrimagnetic bilayer due to the changes in the magnetic moments induced by the interlayer coupling. In general, magnetic Janus heterostructures are expected to comprise rich possibilities for controlling intrinsic magnetic properties.

Figure 5c shows the distribution of all the stable magnetic bilayers according to magnetic order (FM/AFM) and electronic type (metallic/semiconducting). It can be seen that among the semiconductors there is a strong tendency for AFM order while the metals have mainly FM order.

### Ferroelectric switching

Ultrathin vdW materials with bistable stacking configurations could form the basis of ferroelectric devices such as fast non-volatile memories^22–24^. Recent experiments have demonstrated interfacial ferroelectric (IF) switching in bilayers of hBN, some group-VI TMDs, and a few more materials (see ref. ^24^). Beyond those systems IF switching in nine homobilayers of AB honeycomb structures was previously studied using first-principles calculations^45^.

**Fig. 6:**
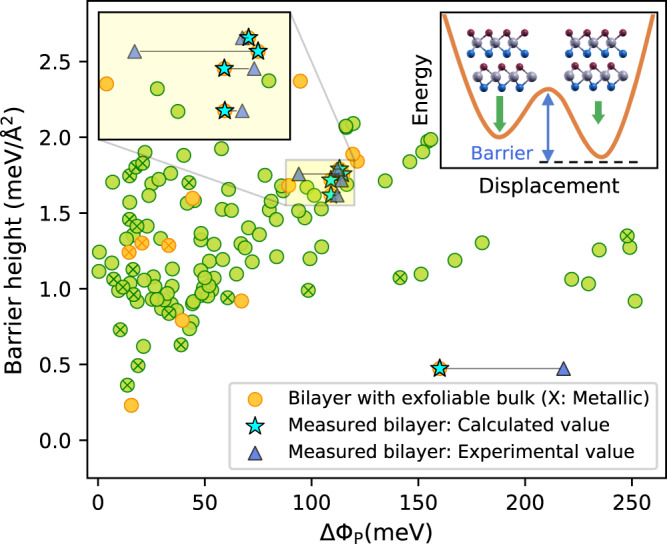
Interfacial ferroelectricity. Calculated energy barrier and polarisation change between pairs of stable stacking configurations related by a pure layer translation for bilayers composed of hexagonal AB, AB_2_, and ABC monolayers. Materials with a known bulk parent are shown in yellow. A cross on a circle indicates that the bilayers are metallic. For the materials for which ferroelectric switching has been experimentally demonstrated (hBN, MoS_2_, MoSe_2_, WS_2_, and WSe_2_), we indicate the calculated (measured) polarisation change with blue stars (triangles). Note that the barrier height values are always calculated as we do not have experimental values available for them. Inset: Sketch of potential energy surface for a bistable bilayer. The green arrows indicate the out-of-plane polarisation in the two stacking configurations.

Our database introduces over 1600 pairs of bilayers with at least two stable stacking configurations related by interlayer translation. To explore the phenomenon of IF more systematically, we have calculated the barrier height and change of out-of-plane polarisation, ΔΦ_P_, for the subset of non-magnetic bistable bilayers composed of hexagonal AB, AB_2_, and ABC monolayers that support two stable and slide-equivalent stacking configurations, see Fig. 6. Within this class we identify 133 bilayer pairs with barriers below 3 meV/Å^2^. These include the experimentally known systems for which our calculated polarisation change, ΔΦ_P_, are in good agreement with the measured values (in meV): 160/218 (hBN), 114/94 (MoS_2_), 108/114 (MoSe_2_), 109/112 (WS_2_), 113/112 (WSe_2_) for calculation/experiment.

As can be seen from Fig. 6, we find several candidates including some that were not studied prior to this work and some with experimentally known bulk phases (yellow symbols). We also highlight the switchable metallic bilayer pairs (cross markers). Interestingly, we find several bilayers composed of ABC Janus monolayers, indicating that the built-in dipole of the monolayer promotes IF. For example, the semiconductors, BiITe, which has been exfoliated in a few-layer form^46^, is bistable with ΔΦ_P_ = 90 meV. Moreover, MoSSe, which has been synthesised as monolayer^47^, exhibits IF in two distinct rotational phases corresponding to twist angles of 0 and 60 degrees and with ΔΦ_P_ of 120 and 146 meV, respectively. A complete overview of all stacking configurations of MoSSe and other selected bilayers can be found in Supplementary section I.

## Discussion

We provided a comprehensive and systematic overview of 8451 unit cell commensurate vdW homobilayers created by combining 1052 monolayers in various stacking configurations. By analysing the stacking orders occurring in 247 natural vdW crystals, we established criteria for bilayers to be stable and experimentally realisable. For 2586 stable bilayers we calculated the electronic band structure, out-of-plane polarisation, interlayer magnetic exchange coupling as well as the Raman tensor for bilayers with up to 8 atoms/unit cell. The low-frequency Raman spectrum was found to be particularly sensitive to the interlayer vdW coupling and to provide a unique fingerprint of the stacking configuration. An abundance of bilayers with emergent properties was identified, e.g., 126 direct band gap semiconductors composed of monolayers with indirect gaps. Moreover, a number of bistable bilayer systems with stacking-dependent polarisation or magnetic order were discovered and proposed as candidates for slidetronics applications. By virtue of their vdW gap and stacking order degree of freedom, the unit cell commensurate homobilayers offer novel functionalities and expand the space of 2D materials beyond the monolayer paradigm.

## Methods

### Workflow and data reproducibility

The computational workflow was constructed within the Atomic Simulation Recipes (ASR) Python framework^48^ and executed using the MyQueue^49^ job scheduler. The ASR workflow employs the GPAW^50^ electronic structure code and the Atomic Simulation Environment (ASE) Python library^51^. All data discussed in this paper can be reproduced by running the ASR workflow.

### Density functional theory calculations

All DFT calculations were performed with the GPAW^50^ code using (unless otherwise stated) a plane wave basis set with cutoff energy 800 eV, a uniform k-point grid of density 12.0/Å^−1^, and a Fermi-Dirac smearing of 50 meV. All monolayers were relaxed using the Perdew-Burke-Ernzerhof (PBE) xc-functional^52^ with spin polarisation. Interlayer distances and binding energies were obtained using the PBE-D3^53^. A minimum vacuum region of 15 Å was used to separate the periodically repeated bilayers.

After structure determination and stability analysis, a static ground state calculation is performed with a tight convergence criterion of 10^−6^ eV/electron. The resulting density is used for the non-selfconsistent calculations of various properties. Spin-orbit coupling is included when evaluating band energies. In the case of magnetic systems, the ground state was calculated for both the FM and AFM interlayer configurations to obtain the most stable (FM/AFM) magnetic state and the size of the interlayer magnetic exchange coupling, *J*.

For all bilayers containing one of the transition metal atoms V, Cr, Mn, Fe, Co, Ni, or Cu, a Hubbard-U correction of 4.0 eV was applied to the 3d orbitals in the calculation of band structures and magnetic properties, if the monolayer was found to exhibit a finite band gap at the PBE+U level. The condition of the monolayer gap is invoked because PBE+U is not justified for systems with metallic screening. In Supplementary Table 1, we show that this approach yields results in good agreement with experiments for a number of known magnetic homobilayers.

### Generation of bilayers

To generate an initial set of possible stacking configurations, we first determine the in-plane rotations that leave the 2D Bravais lattice of the monolayer unchanged but are not symmetry transformations of the monolayer. The set of such rotations, amended by the identity *E*, is denoted by ${{{{{{{\mathcal{R}}}}}}}}$. The rotation axis is always taken to be the *z*-axis, i.e., the origin of the 2D crystal lattice.

All non-oblique Bravais lattices, i.e., all lattices that do not fulfil (*a* ≠ *b* and *γ* ≠ 90^∘^) or (*a* = *b* and *γ* ≠ 90^∘^, 60^∘^, 120^∘^), are invariant under a 180^∘^ rotation around an in-plane axis (flipping). Moreover, the rotation axis can always be taken as the first in-plane basis vector, **a**. This flipping operation is denoted by *F*. For monolayers, which have a non-oblique Bravais lattice but where the monolayer itself is not flip-invariant (i.e., the action of *F* on the monolayer cannot be cancelled by applying an in-plane rotation followed by a translation), we set ${{{{{{{\mathcal{F}}}}}}}}=\{E,\, F\}$. Otherwise we set ${{{{{{{\mathcal{F}}}}}}}}=\{E\}$.

Next, we construct all 2D translation vectors of the form ${{{{{{{{\bf{t}}}}}}}}}_{ij}={{{{{{{{\bf{r}}}}}}}}}_{i}^{\parallel }-{{{{{{{{\bf{r}}}}}}}}}_{j}^{\parallel }$, where ${{{{{{{{\bf{r}}}}}}}}}_{i}^{\parallel }$ is the in-plane position of atom *i* in the monolayer unit cell. The set of such vectors is denoted ${{{{{{{\mathcal{T}}}}}}}}$.

Starting from the AA stacked bilayer, we generate other bilayer structures by applying the transformation(s) ${{{{{{{\mathcal{F}}}}}}}}$ to the lower layer and the transformations $\{{{{{{{{\bf{t}}}}}}}}\circ R\circ F\,| \,F\in {{{{{{{\mathcal{F}}}}}}}},\,R\in {{{{{{{\mathcal{R}}}}}}}},\,{{{{{{{\bf{t}}}}}}}}\in {{{{{{{\mathcal{T}}}}}}}}\}$ to the upper layer. Bilayers, where both lower and upper monolayers have been flipped, are not generated as they are equivalent to the bilayer with both monolayers in the original orientation (by an overall flipping of the entire bilayer). Note that inversion/reflections of the monolayer are not considered, as such transformations may deform the monolayer (for chiral monolayers).

Duplicate structures are removed using a tolerance of 0.6 Å on the distance between identical atoms. The transformations (rotation matrices and translation vectors on the basis of the unit cell vectors) that generate the bilayers from the monolayer are available in the BiDB database. Note that the translation vectors describing the final bilayers may not correspond to any of the initial translation vectors, **t**_*ij*_, as they may be changed by the slide stability workflow (See Supplementary section C).

We stress that the current version of BiDB does not yet contain bilayers where the lower layer has been flipped. Moreover, in some cases, the upper layer was flipped around another in-plane axis than the **a** basis vector. These issues will be resolved in future versions, and we recommend the reader to follow updates on our website.

### Bilayer notation

As described in the previous section, the bilayers are generated by applying affine transformations of the form **t** ∘ *R* ∘ *F* to the upper layer of the AA stacked bilayer and possibly a flip (*F*) of the lower layer. A flip (always around the first basis vector of the 2D unit cell) is indicated by a bar while an in-plane rotation of 2*π*/*n* around the *z*-axis is indicated by a subscript *n*. The translation vector in the 2D unit cell basis may be included in a parenthesis.

Examples of notation: A bilayer obtained by sliding the upper layer by the vector **t** is denoted AB(**t**). The special case AB(0, 0) is denoted AA. A bilayer with the upper layer flipped is denoted ${{{{{{{\rm{A}}}}}}}}\overline{{{{{{{{\rm{B}}}}}}}}}$. A bilayer with the lower layer flipped and the upper layer rotated by 90^∘^ is denoted $\overline{{{{{{{{\rm{A}}}}}}}}}{{{{{{{{\rm{B}}}}}}}}}_{4}$. The 3R and 2H stacking configurations of MoS_2_ are denoted AB(0.33, 0.67) and AB_6_ (0.67, 0.33), respectively.

We note that the notation is partly unit-cell dependent. Specifically, the translation vector **t** obviously depends on the choice of the monolayer unit cell and on the lateral position of the monolayer within the cell. Despite of this ambiguity, the bilayers in the BiDB are uniquely defined by the transformation encoded in our notation when applied to the monolayer structure and unit cell from our own database.

### The *z*-scan approach

For each stacking configuration, we optimise the interlayer distance by minimising the total energy calculated with the PBE-D3 xc-functional^53^ while keeping the monolayers fixed in their PBE-relaxed structure. For non-magnetic systems, a SciPy optimisation was employed to obtain the interlayer distance with a minimal number of DFT calculations. However, for magnetic systems, this approach can lead to unphysical magnetic configurations. Hence, in such cases, we reduce the distance between the monolayers in steps of decreasing size starting from a layer separation of 5 Å (layer separation is here defined as the minimal vertical atom-atom distance). The *z*-scan calculations employed a uniform k-point grid of density 6.0/Å^−1^ and an energy convergence of 10^−4^ eV/electron.

As a byproduct of the *z*-scan approach we obtain a sampling of the binding energy as a function of interlayer distance, *E*_b_(*z*_*n*_). To obtain the exfoliation force we fit *E*_b_(*z*_*n*_) by a Buckingham potential^54^ describing the vdW interactions between two 2D planes. The exfoliation force is then obtained as the maximum of the derivative of the Buckingham potential. We note that for bilayers with large binding energies (i.e., > 30 meV/Å^2^), the Buckingham potential might not be a good fit for the entire binding energy curve. However, since we have more data points in the region around the minimum, the fitted curve is still reliable for estimating the exfoliation force.

The choice of the PBE-D3 xc-functional is motivated by its simplicity, consistency with the PBE used for the monolayer structures, and its relatively good performance for interlayer binding energies and distances in layered materials^55^. We note that the PBE-D3 is expected to work well for bilayers with relatively weak binding energy where the bonding is mainly pure vdW. The majority of the bilayers considered in this work belong to this class. On the other hand, for more strongly bound bilayers, e.g., PtSe_2_ with E_*B*_ = 34 meV/Å^2^, the bonding is of a mixed nature and the PBE-D3 may be less accurate. In Supplementary section A we provide further justification for the choice of xc-functionals and the *z*-scan approach.

### Slide stability

The slide stability of a bilayer is inferred from the local curvature of the 2D potential energy surface (PES). The latter is sampled by a minimum of 8 points obtained by sliding the top layer laterally in steps of 0.15 Å (in some cases step sizes of 0.05 or 0.1 Å were included to obtain an accurate second-order fit). For bilayers corresponding to a saddle point of the PES, a constrained relaxation (with frozen monolayers) was performed until the total force on one monolayer was below 0.01 eV/ Å. The finite-difference sliding test was then repeated to ensure the stability of the generated structure. The computational parameters were the same as those used for the ground state calculations. More details on the slide stability workflow can be found in the Supplementary section C.

### Full relaxation of bilayers

The *z*-scan calculations were supplemented by PBE-D3 calculations in which the bilayers were fully relaxed until the maximum force on any atom was below 0.01 *eV*/ Å. The mean absolute deviation between the interlayer binding energies obtained with the two approaches is only 0.92 meV/Å^2^ (4.3%). The very good agreement shows that intralayer relaxations driven by the layer-layer interactions are weak. We stress that the fully relaxed structures are not necessarily more accurate than the *z*-scan structures, as the PBE-D3 may not be as accurate as the PBE for the strong in-plane bonds. However, for a given bilayer structure of interest it may be relevant to compare the structures and binding energies obtained with the two approaches to assess the effect of interlayer coupling-induced relaxations. More details can be found in the Supplementary section D.

### Raman calculations

First-order Raman spectra were calculated for the non-magnetic bilayers with up to 16 atoms in the unit cell. The calculations are done in the Kramers-Heisenberg-Dirac approximation, where the Raman tensor ${R}_{ij}^{\nu }$ is obtained as the derivative of the electric susceptibility ${\chi }_{ij}^{(1)}$ along the Γ-point phonon modes^56^,

2 $${R}_{ij}^{\nu }=\mathop{\sum}\limits_{\alpha l}\frac{\partial {\chi }_{ij}^{(1)}}{\partial {r}_{\alpha l}}\frac{{v}_{\alpha l}^{\nu }}{\sqrt{{M}_{\alpha }}}.$$

Here, *r*_*α*_ and *M*_*α*_ are the position and atomic mass of atom *α*, respectively, and ${v}_{\alpha l}^{\nu }$ is the eigenmode of phonon *ν*. The phonon modes were calculated using the PBE-D3 xc-functional for a proper description of the vdW-governed interlayer modes (using the Phonopy package). The susceptibility was calculated within the random phase approximation (RPA) using an 800 eV plane wave cutoff and the number of conduction bands was set to twice the number of valence bands. For phonon and susceptibility calculations we used k-point grids with densities of 6 and 15 Å^−1^ respectively. The electric susceptibility tensor, and Raman tensor, are calculated at various important incident wavelengths (488, 532, 780, 980, 1064, 1550) nm with a broadening of 200 meV. The Raman intensity is then calculated for input/output electromagnetic fields with polarisation vectors ${u}_{{{{{{{{\rm{in}}}}}}}}/{{{{{{{\rm{out}}}}}}}}}^{i}$ using

3 $$I(\omega )={I}_{0}\mathop{\sum}\limits_{\nu }\frac{{n}_{\nu }+1}{{\omega }_{\nu }}{\left| \mathop{\sum }\limits_{ij}{u}_{{{{{{{{\rm{in}}}}}}}}}^{i}{R}_{ij}^{\nu }{u}_{{{{{{{{\rm{out}}}}}}}}}^{j}\right| }^{2}\delta (\omega -{\omega }_{\nu }).$$

where *I*_0_ is an unimportant constant, and *n*_*ν*_ is obtained from the Bose–Einstein distribution, i.e., ${n}_{\nu }\equiv {(\exp [\hslash {\omega }_{\nu }/{k}_{B}T]-1)}^{-1}$ at temperature *T* (here set to 300 K) for a Raman mode with energy *ℏω*_*ν*_. The Raman peaks were represented by Gaussians of width 3 cm^−1^ (replacing the delta function in Eq. (3)), which accounts for the spectral broadening of the phonon modes. For more information see^57^.

### NEB calculations

In order to identify possible interfacial ferroelectric bilayers, we start by screening the bilayers for bi-stable pairs (two meta-stable stackings related by a translation). Importantly, we also consider the possibility of a bilayer switching to an in-plane mirror image of itself (e.g., AB/BA stacking of hBN). We then confirm that the bilayer pairs remain slide-switchable after full relaxation. To calculate the barrier heights, we perform regular nudge elastic band (NEB)^58^ calculations followed by a climbing NEB^59^ with a maximum of 7 images along the slide vector between the initial and final stable bilayers. We used BFGS and FIRE as local optimisation algorithms available in ASE for regular and climbing NEB respectively. For these calculations, we employed a uniform k-point grid of density 10.0/Å^−1^ and an energy convergence of 10^−6^ eV/electron and we relaxed the structures until the maximum force on any atom was below 0.01 eV/Å.

### Supplementary information

Supplementary Information

Peer Review File

## Data Availability

The bilayer database will be fully integrated with the C2DB making it easy to explore monolayer and bilayer properties within one coherent framework. The calculated crystal structures and basic properties of the most stable bilayers (binding energy within 10 meV/A^2^ of the most stable stacking configuration), will be made available via the web-application 10.11583/DTU.24849819.

## Code availability

The ASR recipes used in the BiDB workflow are accessible on our website at https://cmr.fysik.dtu.dk/bidb/bidb.html.

## References

[1] Yang H (2022). Two-dimensional materials prospects for non-volatile spintronic memories. Nature.

[2] Sierra JF, Fabian J, Kawakami RK, Roche S, Valenzuela SO (2021). Van der Waals heterostructures for spintronics and opto-spintronics. Nat. Nanotechnol..

[3] Schaibley JR (2016). Valleytronics in 2D materials. Nat. Rev. Mater..

[4] Low T (2017). Polaritons in layered two-dimensional materials. Nat. Mater..

[5] Cao Y (2018). Unconventional superconductivity in magic-angle graphene superlattices. Nature.

[6] Behera B, Sutar BC, Pradhan NR (2021). Recent progress on 2D ferroelectric and multiferroic materials, challenges, and opportunity. Emerg. Mater..

[7] Tran TT, Bray K, Ford MJ, Toth M, Aharonovich I (2016). Quantum emission from hexagonal boron nitride monolayers. Nat. Nanotechnol..

[8] Haastrup S (2018). The computational 2D materials database: high-throughput modeling and discovery of atomically thin crystals. 2D Mater..

[9] Mounet N (2018). Two-dimensional materials from high-throughput computational exfoliation of experimentally known compounds. Nat. Nanotechnol..

[10] Liu X, Pyatakov AP, Ren W (2020). Magnetoelectric coupling in multiferroic bilayer VS2. Phys. Rev. Lett..

[11] Sun Z (2019). Giant nonreciprocal second-harmonic generation from antiferromagnetic bilayer CrI_3_. Nature.

[12] Cenker J (2021). Direct observation of two-dimensional magnons in atomically thin CrI3. Nat. Phys..

[13] Wang S (2021). Two-dimensional ferroelectric channel transistors integrating ultra-fast memory and neural computing. Nat. Commun..

[14] Wu Z (2019). Intrinsic valley hall transport in atomically thin MoS2. Nat. Commun..

[15] Zhang T, Xu X, Huang B, Dai Y, Ma Y (2022). 2D spontaneous valley polarization from inversion symmetric single-layer lattices. npj Comput. Mater..

[16] Leisgang N (2020). Giant stark splitting of an exciton in bilayer MoS2. Nat. Nanotechnol..

[17] Peimyoo, N. et al. Electrical tuning of optically active interlayer excitons in bilayer MoS_2_. *Nat. Nanotechnol*. **16***,* 888–893 (2021). 10.1038/s41565-021-00916-1 34083771

[18] Jiang S, Shan J, Mak KF (2018). Electric-field switching of two-dimensional van der Waals magnets. Nat. Mater..

[19] Huang B (2018). Electrical control of 2D magnetism in bilayer CrI3. Nat. Nanotechnol..

[20] Song T (2019). Switching 2D magnetic states via pressure tuning of layer stacking. Nat. Mater..

[21] Jindal A (2023). Coupled ferroelectricity and superconductivity in bilayer Td-MoTe2. Nature.

[22] Li L, Wu M (2017). Binary compound bilayer and multilayer with vertical polarizations: two-dimensional ferroelectrics, multiferroics, and nanogenerators. ACS Nano.

[23] Yang Q, Wu M, Li J (2018). Origin of two-dimensional vertical ferroelectricity in WTe2 bilayer and multilayer. J. Phys. Chem. Lett..

[24] Wang, C., You, L., Cobden, D. & Wang, J. Towards two-dimensional van der waals ferroelectrics. *Nat. Mater*. **22**, 542–552 (2023). 10.1038/s41563-022-01422-y 36690757

[25] Vizner Stern M (2021). Interfacial ferroelectricity by van der Waals sliding. Science.

[26] Yasuda K, Wang X, Watanabe K, Taniguchi T, Jarillo-Herrero P (2021). Stacking-engineered ferroelectricity in bilayer boron nitride. Science.

[27] Wang X (2022). Interfacial ferroelectricity in rhombohedral-stacked bilayer transition metal dichalcogenides. Nat. Nanotechnol..

[28] Wan Y (2022). Room-temperature ferroelectricity in 1T’-ReS2 multilayers. Phys. Rev. Lett..

[29] Fei Z (2018). Ferroelectric switching of a two-dimensional metal. Nature.

[30] Peng R, Ma Y, Wang H, Huang B, Dai Y (2020). Stacking-dependent topological phase in bilayer MBi2Te4 (M= Ge, Sn, Pb). Phys. Rev. B.

[31] Xu B, Deng J, Ding X, Sun J, Liu JZ (2022). Van der Waals force-induced intralayer ferroelectric-to-antiferroelectric transition via interlayer sliding in bilayer group-IV monochalcogenides. npj Comput. Mater..

[32] Zhou J (2022). Photo-magnetization in two-dimensional sliding ferroelectrics. npj 2D Mater. Appl..

[33] Xiao R-C (2022). Non-synchronous bulk photovoltaic effect in two-dimensional interlayer-sliding ferroelectrics. npj Comput. Mater..

[34] Zhang S (2022). Domino-like stacking order switching in twisted monolayer–multilayer graphene. Nat. Mater..

[35] Shinde SM (2018). Stacking-controllable interlayer coupling and symmetric configuration of multilayered MoS2. NPG Asia Mater..

[36] Bertoldo F (2021). Intrinsic defects in MoS2 grown by pulsed laser deposition: from monolayers to bilayers. ACS Nano.

[37] Gjerding MN (2021). Recent progress of the computational 2D materials database (C2DB). 2D Mater..

[38] Liang L (2017). Low-frequency shear and layer-breathing modes in Raman scattering of two-dimensional materials. ACS Nano.

[39] Mak KF, Lee C, Hone J, Shan J, Heinz TF (2010). Atomically thin MoS2: a new direct-gap semiconductor. Phys. Rev. Lett..

[40] Hong J (2022). Momentum-dependent oscillator strength crossover of excitons and plasmons in two-dimensional PtSe2. ACS Nano.

[41] Li J (2021). Layer-dependent band gaps of platinum dichalcogenides. ACS Nano.

[42] Song T (2018). Giant tunneling magnetoresistance in spin-filter van der Waals heterostructures. Science.

[43] Jiang S, Li L, Wang Z, Shan J, Mak KF (2019). Spin tunnel field-effect transistors based on two-dimensional van der Waals heterostructures. Nat. Electron..

[44] Kruse M (2023). Two-dimensional ferroelectrics from high throughput computational screening. npj Comput. Mater..

[45] Wang Z, Gui Z, Huang L (2023). Sliding ferroelectricity in bilayer honeycomb structures: a first-principles study. Phys. Rev. B.

[46] Fülöp B (2018). Exfoliation of single layer BiTeI flakes. 2D Mater..

[47] Lu A-Y (2017). Janus monolayers of transition metal dichalcogenides. Nat. Nanotechnol..

[48] Gjerding M (2021). Atomic simulation recipes: a Python framework and library for automated workflows. Comput. Mater. Sci..

[49] Mortensen JJ, Gjerding M, Thygesen KS (2020). MyQueue: task and workflow scheduling system. J. Open Source Softw..

[50] Enkovaara J (2010). Electronic structure calculations with GPAW: a real-space implementation of the projector augmented-wave method. J. Phys. Condens. Matter.

[51] Larsen AH (2017). The atomic simulation environment-a Python library for working with atoms. J. Phys. Condens. Matter.

[52] Perdew JP, Burke K, Ernzerhof M (1996). Generalized gradient approximation made simple. Phys. Rev. Lett..

[53] Grimme S, Antony J, Ehrlich S, Krieg H (2010). A consistent and accurate ab initio parametrization of density functional dispersion correction (DFT-D) for the 94 elements H-Pu. J. Chem. Phys..

[54] Li D (2019). From two-to three-dimensional van der Waals layered structures of boron crystals: an ab initio study. ACS Omega.

[55] Tran F, Kalantari L, Traoré B, Rocquefelte X, Blaha P (2019). Nonlocal van der Waals functionals for solids: Choosing an appropriate one. Phys. Rev. Mater..

[56] Lee S-Y, Heller EJ (1979). Time-dependent theory of Raman scattering. J. Chem. Phys..

[57] Taghizadeh A, Leffers U, Pedersen TG, Thygesen KS (2020). A library of ab initio Raman spectra for automated identification of 2D materials. Nat. Commun..

[58] Henkelman G, Jónsson H (2000). Improved tangent estimate in the nudged elastic band method for finding minimum energy paths and saddle points. J. Chem. Phys..

[59] Henkelman G, Uberuaga BP, Jónsson H (2000). A climbing image nudged elastic band method for finding saddle points and minimum energy paths. J. Chem. Phys..

[60] Van Baren J (2019). Stacking-dependent interlayer phonons in 3R and 2H MoS2. 2D Mater..

