# Supplementary material for: High-throughput computational stacking reveals emergent properties in natural van der Waals bilayers
Source: Nat Commun. 2024 Jan 31;15:932. doi: 10.1038/s41467-024-45003-w (PMC10831070; doi:10.1038/s41467-024-45003-w)
Supplement: Supplementary file 1 — Supplementary Information [file 41467_2024_45003_MOESM1_ESM.pdf]

High-throughput computational stacking reveals emergent properties in natural van der Waals bilayers

Sahar Pakdel,[1,*] Asbjørn Rasmussen,[1] Alireza Taghizadeh,[1]
Mads Kruse,[1] Thomas Olsen,[1] and Kristian S. Thygesen[1]

[1] *CAMD, Computational Atomic-Scale Materials Design, Department of Physics, Technical University of Denmark, 2800 Kgs. Lyngby, Denmark.*

* sahpa@dtu.dk

Supplementary Information

Table of Contents

Supplementary Section A Choice of xc-functional for monolayer relaxations 3

Supplementary Section B Exfoliation force 5

Supplementary Section C Slide stability 5

Supplementary Section D z-scan versus full relaxation of bilayers 6

Supplementary Section E Stacking orders in experimental bulk crystals 8

Supplementary Section F Benchmarking of Raman calculations 9

Supplementary Subsection F.1 High frequency intralayer modes in monolayers . . . 9

Supplementary Subsection F.2 Low frequency interlayer modes in bilayers 9

Supplementary Section G Effective exchange constants 11

Supplementary Section H Magnetic bilayers: Comparison to experiments 12

Supplementary Section I Comparison to previous calculations 14

Supplementary Subsection I.1 Content of benchmark tables 14

Supplementary Subsection I.2 Summary of the comparisons 14

Supplementary Section A: Choice of xc-functional for monolayer relaxations

The current work involves calculations with three different xc-functionals: The PBE functional is used to obtain the atomic structure of the 2D monolayers (as they are extracted from the C2DB database). The PBE-D3 is used to obtain the interlayer distance and assess the slide stability (see Supplementary Figure 4). The PBE+U functional is used to calculate the magnetic and electronic properties of the bilayers containing any of the transition metal elements V, Cr, Mn, Fe, Co, Ni, Cu (with $U = 4$ eV for all elements) if the monolayer exhibits a finite band gap at the PBE level. The latter condition is invoked because PBE+U is not justified for systems with metallic screening.

The performance of the three functionals for predicting the in-plane lattice constants of 2D crystals is shown in Supplementary Figure 1. Note that for evaluating the effect of U correction on the lattice constants, specifically for this figure, we applied U correction to the transition metal elements V, Cr, Mn, Fe, Co, Ni, Cu (with U = 3.1, 3.5, 3.8, 4.0, 3.3, 6.4, 4.0 eV respectively as in Ref. [1]). All calculated results refer to a single monolayer while the experimental data refers to the layered bulk parent. All the bulk crystals have weak interactions between the layers as evidenced by the smallness of the calculated interlayer binding energies, which are all below 50 meV/Å^2 (see Fig. 2a in the main text). The justification for comparing in-plane lattice constants of monolayers to such bulk crystals, is provided by Supplementary Figure 2, which shows that the change in the in-plane lattice constant between monolayer and bilayers are negligible (0.37%). This is obviously due to the the weakness of the vdW interlayer coupling in these materials.

From Supplementary Figure 1(a,b,c) we conclude that PBE and PBE-D3 yield accurate and very similar results for in-plane lattice constants (mean absolute deviations (MAD) to experiments of around 1.5% and MAD between the two functionals around 0.6%). It can be seen from panel c, that the inclusion of the D3 term largely corrects the systematic overestimation of lattice constants by PBE, but does so at the price of slightly larger absolute deviations. Based on these results both PBE and PBE-D3 seem as good choices for optimising the 2D layers. We have chosen the PBE due its popular and widespread use, and because it preserves the consistency with the monolayer data in C2DB. The latter makes it easier to compare properties of bilayers in BiDB with monolayer properties in C2DB.

In comparison to PBE and PBE-D3, the PBE+U yields relatively poor in-plane lattice

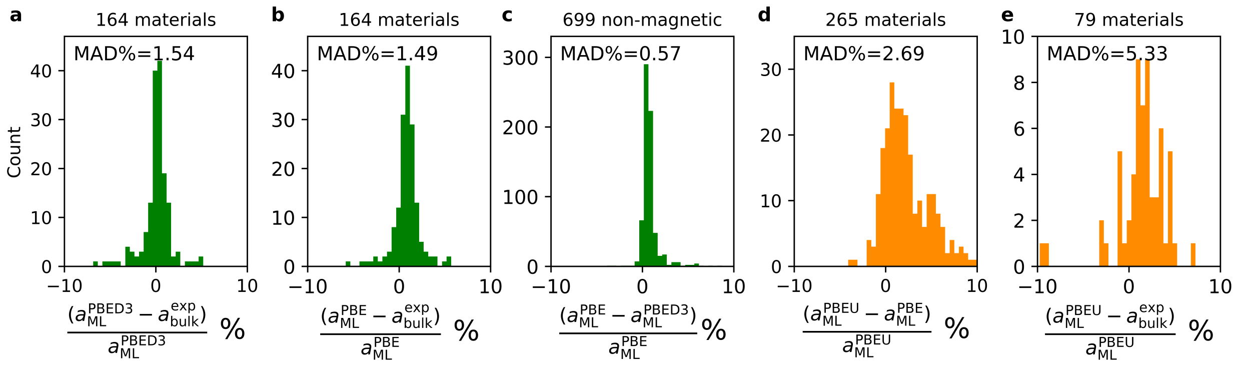

Supplementary Figure 1. **In-plane lattice constants:** Comparison of in-plane lattice constants as estimated by different approximations. The number of materials involved in each analysis is mentioned in the title of each panel. For experimental bulk lattice constants in panels (a, b, e) we use lattice structures from COD and ICSD databases as reference. Panels (d, e) include materials with 3d transition metal atoms to demonstrate the effect of Hubbard-U corrections.

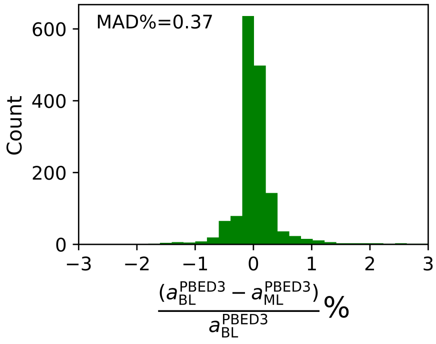

Supplementary Figure 2. **Effect of interlayer interactions on in-plane lattice constants:** Comparison of the in-plane lattice constants of monolayer and bilayer after full relaxation with the PBE-D3 xc-functional. 635 (1618) non-magnetic monolayers (bilayers) are included in this analysis.

constants. Supplementary Figure 1(d,e) show a significant overestimation of lattice constants compared to both PBE (relative MAD of 2.69%) and experiments (5.33%). For this particular set of 79 materials containing one of the transition metal atoms for which we use the U-correction (V, Cr, Mn, Fe, Co, Ni, Cu), the relative MAD of PBE compared to experiments is 2.92%. Based on these results, we have chosen not to perform structural relaxations with PBE+U.

Supplementary Section B: Exfoliation force

To obtain the exfoliation force we fit the binding energies from the z-scan calculations to a Buckingham potential describing the van der Waals energy per area of two infinite planes[2]. Two examples are shown in Supplementary Figure 3. It can be seen that the Buckingham potential provides an accurate description of the PBE-D3 energies over a wide range of interlayer distances. This is generally observed for purely vdW-bonded bilayers (with $E_{athrmb} < 35$ meV/Å^2, approximately). Interestingly, the exfoliation force in the two examples differ significantly while the binding energies are essentially the same. The same conclusion is expressed by Fig. 2b in the main text, and shows that the binding energy alone is not always an accurate descriptor of exfoliability.

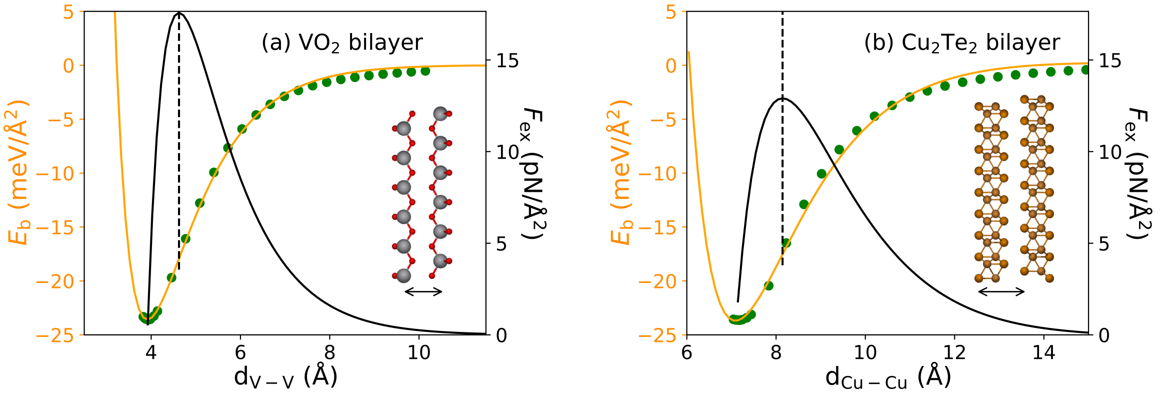

Supplementary Figure 3. **Determining the exfoliation force:** Binding energy curves for two exemplary bilayers. The green dots show PBE-D3 energies while the yellow curve shows a fitted Buckingham potential describing the vdW energy per area between two infinite planes. The black curve shows the derivative of the Buckingham potential and its maximum defines the exfoliation force.

Supplementary Section C: Slide stability

After z-scanning all the generated lateral stacking configurations of a given material, the structures with a binding energy, E_{b} within 3 meV/Å^2 of the most stable configuration, $E_{\mathrm{b,max}}$, are run through the structure validation workflow in order to verify that the structure is stable against sliding.

In case the structure represents a local maximum on the PES, it is not passed on to the property workflow. In case the structure sits on a saddle point, or is offset from a

nearby local minimum, a constrained structure relaxation is performed by moving the upper layer rigidly along the effective force on the layer (the sum of the forces on its constituent atoms). This procedure is repeated until the structure ends up in a local minimum. At this point a full relaxation is performed with the PBE-D3 xc-functional (see next section), and the resulting binding energy, E_b^{relax}, is compared to E_b. In case the difference is below 5 meV/Å^2, the bilayer stacking is considered stable and is passed on to the property workflow.

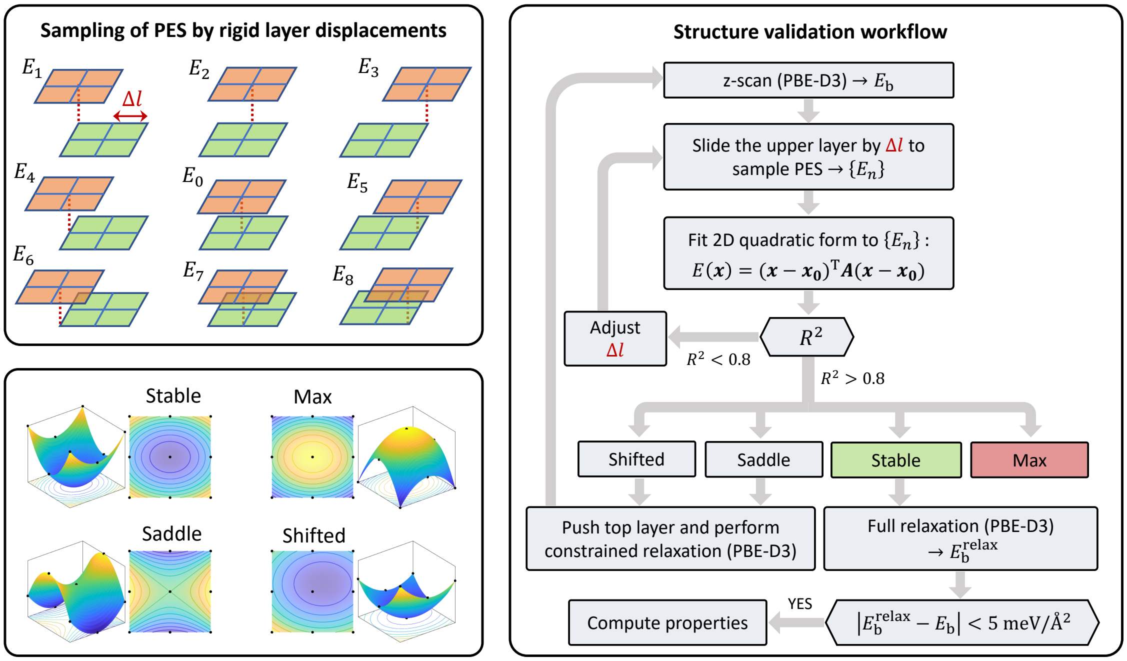

Supplementary Figure 4. **Structure validation workflow:** The right panel shows the workflow used to verify the slide stability of a given bilayer. The lower left panel shows the possible local topologies of the potential energy surfaces (PES) and the upper left panel illustrates how the PES is sampled to determine the topology. In case the structure represents a saddle point of the PES or is shifted relative to a nearby minimum, the upper layer is slided towards the minimum and the procedure is repeated until a slide stable configuration is obtained.

Supplementary Section D: z-scan versus full relaxation of bilayers

In the current work we have employed the z-scan approach to determine the interlayer binding energy and distances of the bilayer structures. In this approach the PBE-D3 functional is used to determine the optimal distance between two PBE-relaxed monolayers placed in a fixed lateral stacking configuration. Thus intralayer relaxations (atomic positions and in-plane lattice constants) induced by the layer-layer interactions, are neglected. To quantify

the importance of such effects we perform full relaxation of the bilayer using the PBE-D3 functional and compare the obtained binding energy to the z-scan results. This step is only performed for slide stable stackings to ensure that the stacking configuration does not change during the relaxation.

Supplementary Figure 5(a) shows the interlayer binding energy obtained from the z-scan method versus a calculation where the binding energy is obtained as the difference between the monolayer and the bilayer where both have been fully relaxed with the PBE-D3 functional. Overall, there is a very good agreement between the two methods. The mean absolute deviation between the interlayer binding energies obtained with the two approaches is only 0.92 meV/Å^2. The binding energies obtained after full relaxation are slightly but systematically larger than the z-scan binding energies. This makes sense as the the relaxations induced by the interlayer coupling should increase the binding energy.

One could expect that the differences in the binding energy obtained with the two approaches would be correlated with the amount of structural changes induced during the full relaxation. However, as seen in Supplementary Figure 5(b,c), ΔE_b is neither correlated with the change in in-plane lattice constant nor the change in layer thickness.

From this analysis we conclude that structural relaxations induced by the layer-layer coupling have minor effects on interlayer binding energy and bilayer structure. This justifies the the z-scan approach. However, binding energies and structures obtained with both the z-scan method and full relaxation are provided in the database.

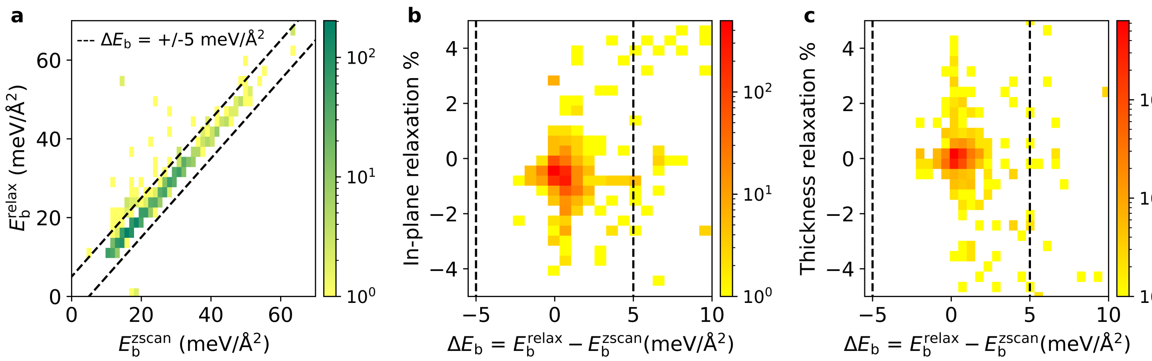

Supplementary Figure 5. **Effect of relaxations** (a) Interlayer binding energy obtained from the z-scan approach versus full relaxation with the PBE-D3 xc-functional. The dashed lines indicate the region where E_b differs by less than 5 meV/Å^2. (b) The difference in the in-plane lattice constant upon relaxation with PBE-D3 and PBE (as used in the z-scan approach) plotted as a function of the difference in interlayer binding energy. (c) Same as (b) but with the change in the thickness along the y-axis.

Supplementary Section E: Stacking orders in experimental bulk crystals

To determine the conditions for a bilayer to be stable and experimentally realisable, we explore the stacking orders in experimentally known layered bulk crystals and verify how far their binding energies are from our predicted most stable bilayer. This analysis allows us to (1) assess how often the predicted most stable bilayer has a stacking order also occurring in the natural bulk crystal (2) to define the $\Delta E_{\mathrm{b,max}} = E_{\mathrm{b,max}} - E_{\mathrm{b}}$ threshold that would capture the majority of naturally occurring stacking orders.

To identify layered vdW crystals we follow Ref. [3] who introduced a dimensionless parameter, s_2, taking values between 0 and 1 as an indicator of the degree to which a bulk crystal can be considered as existing of individual vdW bonded 2D layers. Here, we first identify the layered bulk materials from COD[4] and ICSD[5] with s_2 >0.5 and further filter this set using the same criteria as was used to select the monolayers for the bilayer workflow, i.e the exfoliated monolayer should be dynamically and thermodynamically stable and have a maximum of 10 atoms per unit cell. After this step, we are left with 247 layered bulk crystals. For each bulk crystal, we extract all unique bilayers (crystals with more 2 layers in the primitive unit cell can result in several bilayers) and identify the transformation that generates the bilayer from the monolayer (see "Generation of homobilayers" in Methods).

Since we construct our bilayers based on "relaxed" monolayer structures, the bilayers extracted from the experimental bulk structures are not identical to the bilayers obtained from our computational workflow. This makes the task of matching bilayers from the two sets non-trivial. In particular, there can be small structural differences (that may affect the symmetry) and the in-plane unit cell may differ (i.e. uni-axial or bi-axial strain). For these reasons we avoid using direct structural comparison to match up the experimental and theoretical bilayers. Instead, we define a geometrical "fingerprint" for a bilayer as a radial distribution function for atoms in the lower layer relative to an atom in the upper layer. Such a fingerprint is clearly independent of the chosen unit cell and allows for introducing a tolerance when comparing structures while still being able to discriminate different stacking configurations.

The fingerprint allows us to pair up experimental and theoretical bilayers and thus determine which of the bilayers generated by the computational workflow have stacking configurations also occurring in experimentally known bulk crystals.

Supplementary Section F: Benchmarking of Raman calculations

Raman spectra are calculated using the so-called Kramers–Heisenberg–Dirac method, which obtains the Raman tensor as the derivative of the electric susceptibility with respect to the phonon modes (see Methods section for details).

Supplementary Subsection F.1: High frequency intralayer modes in monolayers

To test the accuracy of the method, we compare the calculated Raman spectra to experimental spectra for nine monolayers, see Supplementary Figure 6. Although the focus of this work is on homobilayers, we first benchmark the method for monolayers as high-accuracy experimental Raman data for bilayers (in well defined stacking configurations) are more scarce.

Across all nine monolayers we see an excellent agreement for the number of active Raman modes and their frequency (deviations below 20 cm^{-1}). In comparison, the relative intensities of the Raman peaks are less accurately reproduced. Overall, the agreement is very satisfactory considering that the substrate interactions (not included in the calculations) can shift Raman peaks[6] and even activate otherwise inactive modes[12].

We mention that the same experimental data was used in our previous work to benchmark a different method for calculating the Raman tensor based on third order perturbation theory in the electron-phonon and light-matter coupling[15]. We find similar accuracy of the two methods when compared to experiments.

Supplementary Subsection F.2: Low frequency interlayer modes in bilayers

In the previous section we benchmarked the accuracy of our Raman calculations for covalently bonded 2D structures. Here we demonstrate that the method also provides an accurate description of the low-frequency Raman spectra of homobilayers, which are governed by the interlayer vdW interactions.

Supplementary Figure 7 shows the comparison of the calculated and measured low-frequency Raman spectra of nine homobilayers. The low-frequency spectrum is determined by the interlayer vibrational modes, which in turn are governed by the weak interfacial vdW

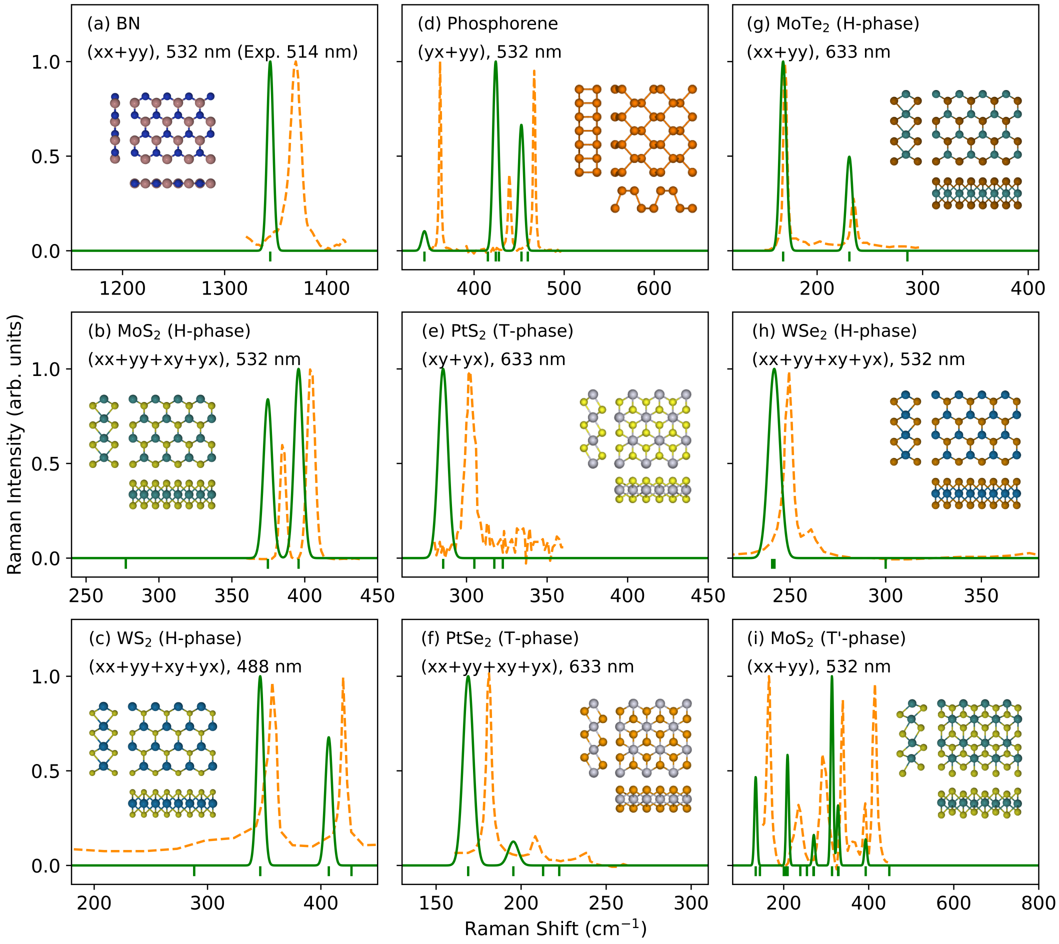

Supplementary Figure 6. **Monolayer Raman spectra.** Comparison of computed Raman spectra (solid lines) with available experimental results (dashed lines) for nine monolayers. The experimental data are extracted from Refs. [6–14] for (a) to (i), respectively. The temperature is set to 300K (room temperature). The excitation wavelength and polarisation components of the Raman tensor are specified for each case. The x- and y-axes lie within the 2D plane.

interactions. In cases where the stacking order is not reported in the experimental reference we have performed the calculation for the most stable stacking. As for the monolayers we see a good qualitative agreement across all materials concerning the number of (active) modes while the absolute deviations in the peak positions below 10 cm^{-1}. We stress that differences in the calculated Raman peak positions/intensities between monolayer and bilayer or between different stacking configurations, are expected to be more accurate than the absolute values.

We note that accurate measurements of low-frequency modes is challenging and requires highly monochromatic lasers to separate the Raman peaks from the elastically scattered light. Moreover, substrate effects, sample purity and/or variations in the stacking pattern

can effect the quality of the experimental spectra. Taking all this into consideration, the agreement between the calculated and experimental spectra is very satisfactory.

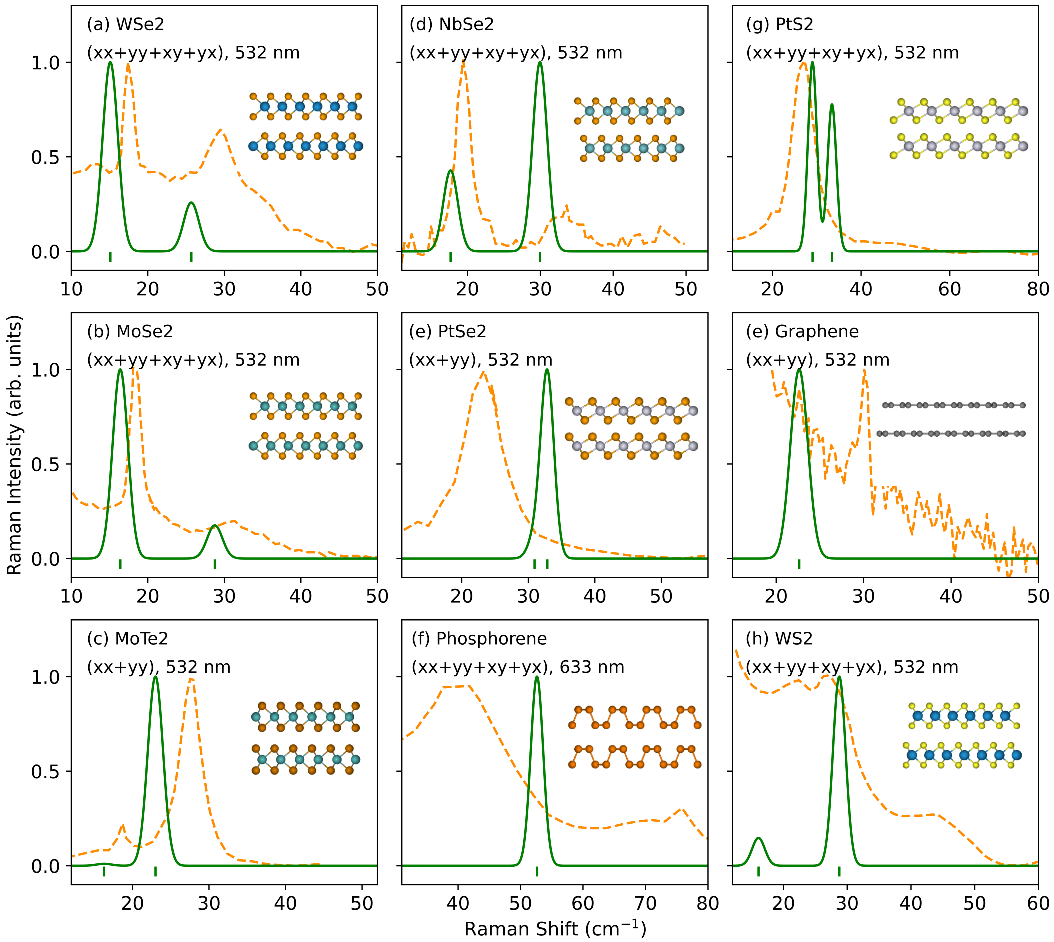

Supplementary Figure 7. **Low frequency Raman spectra.** Comparison of computed low-frequency Raman spectra (solid lines) with available experimental results (dashed lines) for nine homobilayers. The experimental data are extracted from Refs. [11, 14, 16–21] for (a) to (i), respectively. The temperature is set to 300K (room temperature). The excitation wavelength and polarisation components of the Raman tensor are specified for each case. The x- and y-axes lie within the 2D plane.

Supplementary Section G: Effective exchange constants

Magnetic properties are typically modelled in the framework of Heisenberg models and for the bilayers in the present work we consider the model

$$H = \sum_{abij} J_{ij}^{ab} \mathbf{S}_i^a \cdot \mathbf{S}_j^b + H_{\text{intra}}, \tag{G1}$$

where J_{ij}^{ab} are the interlayer exchange constants and H_{intra} contains all intralayer interactions. The sum runs over magnetic atoms a in unit cell i belonging to layer 1 and magnetic atoms b in unit cell j belonging to layer 2. In a classical approximation the energy difference per unit cell between ferromagnetic and anti-ferromagnetic interlayer configurations becomes

$$\Delta E_{\mathrm{mag}} = 2\sum_{abi} S^a S^b J_{0i}^{ab}, \tag{G2}$$

where S^a is the spin (maximal eigenvalue of S^z) of atom a.

In order to retain a unified treatment of interlayer exchange in the present work we define the effective unit cell exchange

$$J = \frac{1}{N_{\mathrm{a}} S^2}\sum_{abi} S^a S^b J_{0i}^{ab}, \qquad S = \frac{1}{N_{\mathrm{a}}}\sum_{a} S^a, \tag{G3}$$

where N_{a} is the number of magnetic atoms in the monolayer and S is the average spin per magnetic atom in the ferromagnetic state of the monolayer. This gives the relation

$$J = \frac{\Delta E_{\mathrm{mag}}}{2N_{\mathrm{a}} S^2} = \frac{N_{\mathrm{a}}\Delta E_{\mathrm{mag}}}{2S_{\mathrm{T}}^2}, \tag{G4}$$

which yields an effective exchange constant that is independent of the unit cell applied for energy mapping. In addition, the introduction of the total spin of the monolayer S_{T} renders the energy mapping independent of assignments of magnetic moments to individual magnetic atoms, which is not a well-defined procedure for metals.

Supplementary Section H: Magnetic bilayers: Comparison to experiments

To benchmark our computational approach for magnetic bilayers and choose an appropriate value for the Hubbard-U term, we compare to available experimental data. Supplementary Table 1 shows the energy difference between the anti-ferromagnetic (AFM) and ferromagnetic (FM) ground states obtained from PBE+U total energy calculations performed on the z-scan bilayer structures. In cases where the experimental reference does not specify the specific stacking order, we consider the most stable stacking configuration predicted by our calculations. The last column shows the experimentally observed magnetic

Material	Bilayer descriptor	U=0	U=3	U=4	U=5	U=6	Reference
CrI_3	AB (0.33, 0.67)	-9.56	-9.59	-9.37	-9.17	-8.86	Exp.[22] FM ($J < 0$)
	AB (0.00, 0.31)	-1.36	1.53	2.91	4.21	-	Exp.[22] AFM ($J > 0$)
$CrCl_3$	AB (0.33, 0.67)	-1.45	-1.16	-1.03	-0.93	-0.81	Exp.[23] AFM ($J > 0$)
CrSBr	AA	-0.15	-0.63	-0.48	-0.32	-0.12	Exp.[24], AFM ($J > 0$)
$NiBr_2$	AA	9.28	3.75	2.76	2.03	1.48	Exp.[25], AFM ($J > 0$)
	AB (0.67, 0.33)	9.26	3.75	2.85	2.07	1.52	Exp.[25], AFM ($J > 0$)
$NiCl_2$	AA	3.95	1.56	1.15	0.84	0.63	Exp.[25], AFM ($J > 0$)
$CoCl_2$	AA	3.74	1.33	0.98	0.77	0.58	Exp.[25], AFM ($J > 0$)
$CoBr_2$	AA	8.09	2.81	2.06	1.52	1.14	Exp.[25], AFM ($J > 0$)

Supplementary Table 1. **Relative stability of magnetic states.** The table shows the energy difference (in meV/$Å^2$) between the anti-ferromagnetic (AFM) and ferromagnetic (FM) states for different bilayers. A negative number implies FM order. Results are shown for different values of the Hubbard-U term used in the PBE+U calculations. The considered stacking configuration is specified by the bilayer descriptor in the second column. For CrI_3 and $NiBr_2$ the stacking configurations are listed in order of their stability (most stable first). For all other materials the most stable configuration is shown. The last column shows the experimentally observed magnetic order (in the low temperature phase where available).

state (sign of the interlayer exchange coupling).

The experimentally most well characterised material is CrI_3. For this material all Hubbard-U values above 2 eV yield qualitatively correct result. The predicted most stable stacking configuration (upper row), which corresponds to the low temperature phase and is the stacking found in natural CrI_3 crystals, has a FM ground state while the second most stable stacking configuration (lower row), which corresponds to the high temperature phase, has an AFM ground state[22]. In contrast, the pure PBE result (U=0) predicts FM ground states for both stackings.

For $CrCl_3$ and CrSBr we predict a FM ground state for all U-values. This is in disagreement with experiments, which observe AFM order, but in agreement with other previous DFT calculations[26]. We note that both materials, in particular CrSBr, have weak exchange coupling, making the prediction of the correct ordering of the magnetic states particularly challenging. For the remaining four MX_2 transition metal dihalides, we obtain AFM order for all U-values in agreement with the experimental results.

Based on these results, we argue that the precise value of U does not have a large effect on the relative stability of FM and AFM states as obtained from first principles. Hence, we have chosen to use a constant, i.e. element independent, U-value of 4.0 eV following the

principle of Occam's razor.

Supplementary Section I: Comparison to previous calculations

This section contains tables comparing the bilayers generated by our workflow (in blue) with computational studies from the literature (in black). The methods used in the reference studies are summarised in the caption of each table. Note that our workflow treats all monolayers on the same footing and is not restricted to specific cell/crystal symmetries.

Supplementary Subsection I.1: Content of benchmark tables

The tables are organised as follows: The first columns (Stacking order) provide information about the stacking order using our notation (blue) and the notation from the reference (black). Next, the interlayer binding energies are reported together with the order of stability of the stacking configurations (from the most stable to the least stable). Then the in-plane lattice constant, a (for in-plane anisotropic materials only the smallest), and interlayer distances are listed. The interlayer distances are given as the center-to-center distance (d_{cen}) or the minimum vertical distance between atoms in different layers (d_{min}), depending on what is reported in the reference article. For magnetic bilayers we also report the most stable magnetic order (FM or AFM) and the energy difference per unit cell between the two ($\Delta E_{\mathrm{mag}} = E_{\mathrm{FM}} - E_{\mathrm{AFM}}$). Fields with '-' indicates that the relevant information is missing from the reference. In the Table 2 we show an example of the benchmark tables for MoS_2 in the H-phase. We have marked the slide stable (local minimum of the PES) and thermodynamically stable materials (defined to be within 3 meV/Å^2 of the most stable stacking configuration) by a green background. For this example, we show all the bilayers generated by the workflow. In the rest of the benchmark tables we will for simplicity only show the stable stackings. However, all the generated bilayers can be found on our database.

Supplementary Subsection I.2: Summary of the comparisons

For the non-magnetic bilayers we generally find excellent agreement with previous results. The small differences found are most likely due to differences in the employed methodology, in particular the difference xc-functionals. A few cases call for explanation.

**Homobilayers of MoS_2 (H-phase)**									
Stacking order		E_b [meV/Å^2]		Stability		a [Å]		d_{cen} [Å]	
AB(0.33, 0.67)	AB'	26.68	15.31	1	3	3.18	3.16	6.17	6.27
AB_6 (0.67, 0.33)	AA'	26.66	17.14	2	1	3.18	3.16	6.19	6.24
AB_6 (0.00, 0.00)	AB	25.18	16.91	3	2	3.18	3.16	6.24	6.17
AB (0.50, 0.00)		24.93		4		3.18		6.27	
AB_6 (0.50, 0.00)		21.84		5		3.18		6.49	
AB (0.83, 0.67)		21.59		6		3.18		6.50	
AB_6 (0.17, 0.33)		21.54		7		3.18		6.50	
AB_6 (0.33, 0.67)	A'B	19.32	10.71	8	4	3.18	3.16	6.71	6.84
AA	AA	18.95	10.36	9	5	3.18	3.16	6.74	6.87

Supplementary Table 2. Comparison of our results (blue) with results from Ref. [27] (black), which were obtained using VASP with the PBE functional and D2 van der Waals corrections. In the reference, the in-plane lattice constant was taken as the experimental one for bulk. Bilayers predicted to be stable (slide stable and binding energy within 3 meV/Å^2 of the most stable stacking configuration) by our workflow are indicated by a green background. Note that several of the stackings generated by our workflow were not studied in the reference paper.

For the four group-VI TMDs (MX_2, M=Mo, W, X=S, Se) we find the AB' structure (AB in our notation) to be more stable than the AB structure (AB_2 in our notation) whereas Ref. [27] reports the opposite. In addition, for MoS_2 we find the AB' structure (AB in our notation) to be the most stable whereas Ref. [27] predicts the AA' (AB_2 in our notation) to be the most stable. The energy difference between these structures is, however, a mere 0.01 meV/Å^2. These differences are likely due to the different xc-functionals used, namely PBE+D3 (present work) and PBE+D2 (reference) or the choice of in-plane lattice constants, which were chosen as the experimental values in the reference.

For bilayers of the two Janus structures MoSSe and WSSe our algorithm for generating bilayers find all the structures considered in Ref. [28] and Ref. [29], respectively. However, we also find an additional highly stable structure (within 1.5 meV/Å^2 of the most stable structure) for both Janus bilayers that was not considered in the references. Apparently the authors missed the fact that the AB structures (in the notation of the references) SMSe-SMSe and SeMS-SeMS (with M=Mo, W) are distinct, which is perhaps not so obvious from simple considerations of stacking possibilities. This highlights the advantage of the systematic approach to bilayer generation applied in the present work.

For most of the magnetic materials we also find good agreement with previous theoretical estimates both for the relative stability of different stacking orders and for the interlayer

magnetic order. The most extensively studied magnetic bilayer is comprised by CrI_3, where the bulk high temperature stacking order (C2/m) is retained in bilayers cooled below the ordering temperature. The high temperature phase exhibits AFM interlayer order whereas another low temperature bulk phase ($R\bar{3}$) constitutes the global minimum with FM interlayer order. For the the isostructural material $CrBr_3$, we also reproduce previous theoretical estimates for the magnetic order (obtained with PBE+U). The predicted AFM interlayer order of $CrBr_3$ appears to be in agreement with experiments. In case of $CrCl_3$, however, we couldn't converge the FM and AFM calculations with enough accuracy to compare with the interlayer order with experimental reports of AFM interlayer order [30].

The most significant deviations from previous theoretical results are constituted by bilayers of CrBrS. Where the interlayer exchange energy is exceedingly small (~ 0.5 meV per unit cell) and could be rather sensitive to the choice of lattice parameters and choice of functional for the geometry relaxation.

**Homobilayers of $MoSe_2$ (H-phase)**									
Stacking order		E_b [meV/Å^2]		Stability		a [Å]		d_{cen} [Å]	
AB_6 (0.67, 0.33)	AA'	28.61	22.81	1	1	3.32	3.29	6.52	6.53
AB (0.33, 0.67)	AB'	28.43	20.44	2	3	3.32	3.29	6.50	6.63
AB_6 (0.00, 0.00)	AB	26.65	22.52	3	2	3.32	3.29	6.59	6.53

Supplementary Table 3. Comparison of our results (blue) with results from Ref. [27] (black), which were obtained using VASP with the PBE functional and D2 van der Waals corrections. The in-plane lattice constant was taken as the experimental one for bulk. Here we show 3 out of 9 studied bilayer stackings. The bilayers with slide instability or binding energies weaker than 3 meV/Å^2 compared to the most stable are not shown. The full information is available on our database.

**Homobilayers of WS_2 (H-phase)**									
Stacking order		E_b [meV/Å^2]		Stability		a [Å]		d_{cen} [Å]	
AB_6 (0.67, 0.33)	AA'	27.67	21.02	1	1	3.19	3.15	6.24	6.24
AB (0.33, 0.67)	AB'	27.37	18.82	2	3	3.19	3.15	6.23	6.24
AB_6 (0.00, 0.00)	AB	25.78	20.65	3	2	3.19	3.15	6.32	6.24

Supplementary Table 4. Comparison of our results (blue) with results from Ref. [27] (black), which were obtained using VASP with the PBE functional and D2 van der Waals corrections. The in-plane lattice constant was taken as the experimental one for bulk. Here we show 3 out of 9 studied bilayer stackings. The bilayers with slide instability or binding energies weaker than 3 meV/Å^2 compared to the most stable are not shown. The full information is available on our database.

**Homobilayers of WSe_2 (H-phase)**									
Stacking order		E_b [meV/Å^2]		Stability		a [Å]		d_{cen} [Å]	
AB_6 (0.67, 0.33)	AA'	30.91	26.99	1	1	3.32	3.28	6.51	6.54
AB (0.33, 0.67)	AB'	30.45	23.99	2	3	3.32	3.28	6.52	6.59
AB_6 (0.00, 0.00)	AB	28.42	26.33	3	2	3.32	3.28	6.62	6.54

Supplementary Table 5. Comparison of our results (blue) with results from Ref. [27] (black), which were obtained using VASP with the PBE functional and D2 van der Waals corrections. The in-plane lattice constant was taken as the experimental one for bulk. Here we show 3 out of 9 studied bilayer stackings. The bilayers with slide instability or binding energies weaker than 3 meV/Å^2 compared to the most stable are not shown. The full information is available on our database.

**Homobilayers of MoSSe (H-phase)**									
Stacking order		E_b [meV/Å^2]		Stability		a [Å]		d_min [Å]	
$\overline{\mathrm{A}}\mathrm{B}_6$ (0.67, 0.33)	AA'$_{SeSe}$	29.01	29.09	1	1	3.25	-	3.21	3.21
$\overline{\mathrm{A}}\mathrm{B}$ (0.33, 0.67)	AB$_{SeSe}$	28.41	28.47	2	3	3.25	-	3.23	3.24
AB (0.67, 0.33)	AB$_{SSe}$	28.22	28.54	3	2	3.25	-	3.07	3.12
AB$_6$ (0.67, 0.33)	AA'$_{SSe}$	27.98	28.31	4	4	3.25	-	3.11	3.14
AB (0.33, 0.67)	-	27.55	-	5	-	3.25	-	3.11	-
$\mathrm{A}\overline{\mathrm{B}}$ (0.33, 0.67)	AB$_{SS}$	26.90	26.96	6	5	3.25	-	2.99	3.03
$\mathrm{A}\overline{\mathrm{B}}_6$ (0.67, 0.33)	AA'$_{SS}$	26.44	26.54	8	6	3.25	-	3.04	3.08
$\overline{\mathrm{A}}\mathrm{B}_6$ (0.00, 0.00)	AB'$_{SeSe}$	26.41	-	9	-	3.25	-	3.34	-
AB$_6$ (0.00, 0.00)	AB'$_{SSe}$	26.25	-	10	-	3.25	-	3.17	-

Supplementary Table 6. Comparison of our results (blue) with results from Ref. [28] (black), which were obtained using VASP with the PBE functional and D2 van der Waals corrections. All structures in the reference were fully relaxed and the optimized lattice constant for the monolayer was used for bilayer calculations. Here we show 9 out of 32 studied bilayer stackings. The bilayers with slide instability or binding energies weaker than 3 meV/Å^2 compared to the most stable are not shown. The full information is available on our database.

**Homobilayers of WSSe (H-phase)**									
Stacking order		E_b [meV/Å^2]		Stability		a [Å]		d_cen [Å]	
$\overline{\mathrm{A}}\mathrm{B}_6$ (0.67, 0.33)	AA'$_{SSeSeS}$	31.37	35.34	1	1	3.25	3.25	6.61	6.52
$\overline{\mathrm{A}}\mathrm{B}$ (0.33, 0.67)	AB$_{SSeSeS}$	30.51	34.24	2	2	3.25	3.25	6.64	6.53
AB$_6$ (0.67, 0.33)	AA'$_{SeSSeS}$	30.13	33.17	3	3	3.25	3.25	6.34	6.25
AB (0.67, 0.33)	-	29.86	-	4	-	3.25	-	6.32	-
AB (0.33, 0.67)	AB$_{SeSSeS}$	28.99	32.34	5	4	3.25	3.25	6.38	6.25

Supplementary Table 7. Comparison of our results (blue) with results from Ref. [29] (black), which were obtained using VASP with the PBE functional and D2 van der Waals corrections. All structures in the reference were fully relaxed and the optimized lattice constant for the monolayer was used for bilayer calculations. Here we show 5 out of 30 studied bilayer stackings. The bilayers with slide instability or binding energies weaker than 3 meV/Å^2 compared to the most stable are not shown. The full information is available on our database.

**Homobilayers of SnS_2 (T-phase)**									
Stacking order		E_b [meV/Å^2]		Stability		a [Å]		d_min [Å]	
AA	AA	16.22	-	1	1	3.70	3.68	3.02	2.95
AB$_6$ (0.67, 0.33)	A'B	16.04	-	2	2	3.70	3.68	3.03	2.97
AB (0.67, 0.33)	AB	15.23	-	3	3	3.70	3.68	3.10	3.03

Supplementary Table 8. Comparison of our results (blue) with results from Ref. [31] (black), which were obtained using VASP with the PBE functional and D2 van der Waals corrections. The bilayer structures were fully optimized. Here we show 3 out of 10 studied bilayer stackings. The bilayers with slide instability or binding energies weaker than 3 meV/Å^2 compared to the most stable are not shown. The full information is available on our database.

**Homobilayers of P_4**									
Stacking order		E_b [meV/Å^2]		Stability		a [Å]		d_{min} [Å]	
AB (1.00, 0.50)	AB	21.45	-	1	1	4.63	4.52	3.23	3.58
AB (0.28, 1.00)	-	19.61	-	2	-	4.63	-	3.30	-
AB (0.72, 1.00)	-	19.61	-	2	-	4.63	-	3.30	-

Supplementary Table 9. Comparison of our results (blue) with results from Ref. [32] (black), which were obtained using Quantum Espresso with the PBE functional and vdW-DF van der Waals corrections. The bilayer structures were fully optimized. Here we show 3 out of 21 studied bilayer stackings. The bilayers with slide instability or binding energies weaker than 3 meV/Å^2 compared to the most stable are not shown. The full information is available on our database.

**Homobilayers of h-BN**									
Stacking order		E_b [meV/Å^2]		Stability		a [Å]		d_{cen} [Å]	
AB_6 (0.33, 0.67)	AA'	16.229	-	1	1	2.51	2.52	3.44	3.13
AB (0.67, 0.33)	AB	16.11	-	2	2	2.51	2.52	3.42	3.13
AB_6 (0.00, 0.00)	AB'	15.35	-	3	3	2.51	2.52	3.48	3.13

Supplementary Table 10. Comparison of our results (blue) with results from Ref. [33] (black), which were obtained using VASP with the PBE functional and D van der Waals corrections. The bilayer structures in the reference were fully optimized. Here we show 3 out of 11 studied bilayer stackings. The bilayers with slide instability or binding energies weaker than 3 meV/Å^2 compared to the most stable are not shown. The full information is available on our database.

**Homobilayers of h-BN**									
Stacking order		E_b [meV/Å^2]		Stability		a [Å]		d_{cen} [Å]	
AB_6 (0.33, 0.67)	AA'	16.229	14.03	1	2	2.51	2.50	3.44	3.37
AB (0.67, 0.33)	-	16.11	-	2	-	2.51	-	3.42	-
AB_6 (0.00, 0.00)	AB_1	15.35	14.29	3	1	2.51	2.50	3.48	3.37

Supplementary Table 11. Comparison of our results (blue) with results from Ref. [34] (black), which were obtained using FHI with the PBE functional and TS van der Waals corrections. The lattice constant was taken as the experimental bulk value. Here we show 3 out of 11 studied bilayer stackings. The bilayers with slide instability or binding energies weaker than 3 meV/Å^2 compared to the most stable are not shown. The full information is available on our database.

**Homobilayers of $PtSe_2$ (T-phase)**									
Stacking order		E_b [meV/Å^2]		Stability		a [Å]		d_{cen} [Å]	
AA	AA_1	34.26	26.09	1	1	3.75	3.74	4.92	5.14

Supplementary Table 12. Comparison of our results (blue) with results from Ref. [35] (black), which were obtained using VASP with the PBE functional and optB86b van der Waals corrections. The bilayer structures were fully optimized. Here we show 1 out of 5 studied bilayer stackings. The bilayers with slide instability or binding energies weaker than 3 meV/Å^2 compared to the most stable are not shown. The full information is available on our database.

**Homobilayers of Cr_2I_6**												
Stacking order		E_b [meV/Å^2]		Stability		a [Å]		d_{cen} [Å]		Mag. order		ΔE_{mag}
AB (0.33, 0.67)	AB	13.74	-	1	1	7.01	6.89	6.67	6.68	FM	FM	-9.37
AB (0.00, 0.31)	AB'	13.56	-	2	2	7.01	6.89	6.69	6.73	AFM	AFM	2.91
AB_6 (0.67, 0.02)	-	13.46	-	3	-	7.01	-	6.70	-	FM	-	-3.89
AA	-	12.34	-	4	-	7.01	-	6.82	-	FM	-	-1.52

Supplementary Table 13. Comparison of our results (blue) with results from Ref. [36] (black), which were obtained using VASP with the PBEsol+U functional (U=3 eV on Cr *d*-orbitals) and D3 van der Waals corrections. The bilayer structures were fully optimized in the reference. Here we show 4 out of 37 studied bilayer stackings. The bilayers with slide instability or binding energies weaker than 3 meV/Å^2 compared to the most stable are not shown. The full information is available on our database.

**Homobilayers of Cr_2Br_6**												
Stacking order		E_b [meV/Å^2]		Stability		a [Å]		d_{cen} [Å]		Mag. order		ΔE_{mag}
AB (0.33, 0.67)	AB	13.44	-	1	1	6.45	-	6.14	-	FM	FM	-2.57
AB (0.00, 0.32)	HT	13.22	-	2	2	6.45	-	6.18	-	AFM	AFM	0.26
AB_6 (0.67, 0.02)	rHT'	13.19	-	3	3	6.45	-	6.17	-	FM	FM	-1.47
AA	AA	12.41	-	4	4	6.45	-	6.26	-	FM	AFM	-0.70

Supplementary Table 14. Comparison of our results (blue) with results from Ref. [37] (black), which were obtained using Quantum Espresso with the vdW-DF2 functional. The monolayers were relaxed using experimental lattice constants and kept fixed while interlayer distances were varied in the bilayers. Here we show 4 out of 23 studied bilayer stackings. The bilayers with slide instability or binding energies weaker than 3 meV/Å^2 compared to the most stable are not shown. The full information is available on our database.

**Homobilayers of Cr_2Cl_6**												
Stacking order		E_b [meV/Å^2]		Stability		a [Å]		d_{cen} [Å]		Mag. order		ΔE_{mag}
AB (0.33, -0.33)	AB	12.42	-	1	1	6.06	-	5.80	-	-	AFM	-
AB (0.32, 0.00)	HT	12.28	-	2	2	6.06	-	5.84	-	-	AFM	-
AB_6 (0.32, 0.00)	rHT	12.25	-	3	3	6.06	-	5.82	-	-	AFM	-
AA	AA	11.74	-	4	4	6.06	-	5.90	-	-	AFM	-

Supplementary Table 15. Comparison of our results (blue) with results from Ref. [37] (black), which were obtained using Quantum Espresso with the vdW-DF2 functional. The monolayers were relaxed using experimental lattice constants and kept fixed while interlayer distances were varied in the bilayers. Here we show 4 out of 25 studied bilayer stackings. The bilayers with slide instability or binding energies weaker than 3 meV/Å^2 compared to the most stable are not shown. The full information is available on our database.

**Homobilayers of $Cr_2Br_2S_2$**												
Stacking order		E_b [meV/Å^2]		Stability		a [Å]		d_{cen} [Å]		Mag. order		ΔE_{mag}
AA	AA	16.79	-	1	1	4.74	4.70	8.06	8.10	FM	AFM	-0.48

Supplementary Table 16. Comparison of our results (blue) with results from Ref. [38] (black), which were obtained using Quantum Espresso with the PBE functional and D2 van der Waals corrections. The bilayer structures in the reference were fully optimized. Here we show 1 out of 9 studied bilayer stackings. The bilayers with slide instability or binding energies weaker than 3 meV/Å^2 compared to the most stable are not shown. The full information is available on our database.

**Homobilayers of VSe_2 (H-phase)**												
Stacking order		E_b [meV/Å^2]		Stability		a [Å]		d_{cen} [Å]		Mag. order		ΔE_{mag}
AB (0.33, 0.67)	-	26.59	-	1	-	3.34	-	6.38	-	FM	-	-0.80
AB_6 (0.00, 0.00)	A'B	26.3	-	2	1	3.34	-	6.37	-	AFM	AFM	0.72
AB_6 (0.67, 0.33)	AB	26.3	-	3	2	3.34	-	6.42	-	AFM	AFM	0.43

Supplementary Table 17. Comparison of our results (blue) with results from Ref. [39] (black), which were obtained using VASP with the PBE+U functional (U=1 eV on V d-orbitals) and D2 van der Waals corrections. The bilayer structures were fully optimized in the reference. Here we show 3 out of 10 studied bilayer stackings. The bilayers with slide instability or binding energies weaker than 3 meV/Å^2 compared to the most stable are not shown. The full information is available on our database.

**Homobilayers of $CoBr_2$ (T-phase)**												
Stacking order		E_b [meV/Å^2]		Stability		a [Å]		d_{cen} [Å]		Mag. order		ΔE_{mag}
AA	AA	13.73	-	1	1	3.73	3.74	6.15	-	-	AFM	-
AB (0.67, 0.33)	AB	13.18	-	2	2	3.73	-	6.18	-	AFM	AFM	1.86

Supplementary Table 18. Comparison of our results (blue) with results from Ref. [40] (black), which were obtained using VASP with the PBE+U functional (U=3 eV on Co d-orbitals) and optB86b van der Waals corrections. The bilayer structures were fully optimized in the reference. Here we show 2 out of 10 studied bilayer stackings. The bilayers with slide instability or binding energies weaker than 3 meV/Å^2 compared to the most stable are not shown. The full information is available on our database.

**Homobilayers of $NiBr_2$ (T-phase)**												
Stacking order		E_b [meV/Å^2]		Stability		a [Å]		d_{cen} [Å]		Mag. order		ΔE_{mag}
AA	AA	13.93	-	1	2	3.70	-	6.10	-	AFM		2.79
AB_6 (0.67, 0.33)	AB	13.87	-	2	1	3.70	3.70	6.11	-	AFM	FM	2.34
AB (0.67, 0.33)	-	13.72	-	3	-	3.70	-	6.11	-	AFM	-	2.81

Supplementary Table 19. Comparison of our results (blue) with results from Ref. [41] (black), which were obtained using VASP with the PBE+U functional (U=4 eV on Ni d-orbitals) and D3 van der Waals corrections. The bilayer structures were fully optimized in the reference. Here we show 3 out of 10 studied bilayer stackings. The bilayers with slide instability or binding energies weaker than 3 meV/Å^2 compared to the most stable are not shown. The full information is available on our database.

**Homobilayers of $NiCl_2$ (T-phase)**												
Stacking order		E_b [meV/Å^2]		Stability		a [Å]		d_{cen} [Å]		Mag. order		ΔE_{mag}
AA	AA	12.69	-	1	2	3.50	-	5.76	-	AFM	-	1.20
AB_6 (0.67, 0.33)	-	12.61	-	2	-	3.50	-	5.77	-	AFM	-	0.95
AB (0.67, 0.33)	AB	12.49	-	3	1	3.50	3.49	5.79	-	AFM	FM	1.14

Supplementary Table 20. Comparison of our results (blue) with results from Ref. [41] (black), which were obtained using VASP with the PBE+U functional (U=4 eV on Ni d-orbitals) and D3 van der Waals corrections. The bilayer structures were fully optimized. Here we show 3 out of 10 studied bilayer stackings. The bilayers with slide instability or binding energies weaker than 3 meV/Å^2 compared to the most stable are not shown. The full information is available on our database.

**Homobilayers of NiI_2 (T-phase)**												
Stacking order		E_b [meV/Å^2]		Stability		a [Å]		d_{cen} [Å]		Mag. order		ΔE_{mag}
AB_6 (0.67, 0.33)	-	14.85	-	1	-	3.97	-	6.61	-	AFM	-	5.15
AA	AA	14.80	-	2	2	3.97	-	6.60	-	AFM	-	6.36
AB (0.67, 0.33)	AB	14.69	-	3	1	3.97	4.01	6.62	-	AFM	FM	6.56

Supplementary Table 21. Comparison of our results (blue) with results from Ref. [41] (black), which were obtained using VASP with the PBE+U functional (U=4 eV on Ni d-orbitals) and D3 van der Waals corrections. The bilayer structures were fully optimized. Here we show 3 out of 10 studied bilayer stackings. The bilayers with slide instability or binding energies weaker than 3 meV/Å^2 compared to the most stable are not shown. The full information is available on our database.

**Homobilayers of Bi_2MnTe_4**												
Stacking order		E_b [meV/Å^2]		Stability		a [Å]		d_{cen} [Å]		Mag. order		ΔE_{mag}
AB (0.67, 0.33)	-	21.29	-	1	1	4.34	-	13.66	-	AFM	AFM	0.81
AB_6 (0.33, 0.67)	-	20.77	-	2	-	4.34	-	13.85	-	AFM	-	0.44
AB (0.33, 0.67)	-	19.24	-	3	-	4.34	-	14.04	-	-	-	-

Supplementary Table 22. Comparison of our results (blue) with results from Ref. [42] (black), which were obtained using VASP with the PBE+U functional (U=4 eV on Mn d-orbitals) and D3 van der Waals corrections. The bilayer structures were fully optimized. Here we show 3 out of 10 studied bilayer stackings. The bilayers with slide instability or binding energies weaker than 3 meV/Å^2 compared to the most stable are not shown. The full information is available on our database.

Supplementary references

1. Wang, L., Maxisch, T. & Ceder, G. Oxidation energies of transition metal oxides within the GGA+U framework. Physical Review B **73,** 195107 (2006).
2. Li, D. et al. From two-to three-dimensional van der waals layered structures of boron crystals: an ab initio study. ACS omega **4,** 8015–8021 (2019).
3. Larsen, P. M., Pandey, M., Strange, M. & Jacobsen, K. W. Definition of a scoring parameter to identify low-dimensional materials components. Physical Review Materials **3,** 034003 (2019).
4. Gražulis, S. et al. Crystallography Open Database (COD): an open-access collection of crystal structures and platform for world-wide collaboration. Nucleic acids research **40,** D420–D427 (2012).
5. Belsky, A., Hellenbrandt, M., Karen, V. L. & Luksch, P. New developments in the Inorganic Crystal Structure Database (ICSD): accessibility in support of materials research and design. Acta Crystallographica Section B: Structural Science **58,** 364–369 (2002).
6. Cai, Q. et al. Raman signature and phonon dispersion of atomically thin boron nitride. Nanoscale **9,** 3059–3067 (2017).
7. Xia, F., Wang, H. & Jia, Y. Rediscovering black phosphorus as an anisotropic layered material for optoelectronics and electronics. Nature Communications **5,** 4458 (2014).
8. Yang, M. et al. Anharmonicity of monolayer MoS2, MoSe2, and WSe2: A Raman study under high pressure and elevated temperature. Applied Physics Letters **110,** 093108 (2017).
9. Ruppert, C., Aslan, B. & Heinz, T. F. Optical properties and band gap of single-and few-layer MoTe2 crystals. Nano Letters **14,** 6231–6236 (2014).
10. Liu, H.-L. et al. Deep-ultraviolet Raman scattering spectroscopy of monolayer WS2. Scientific Reports **8,** 11398 (2018).
11. Zhao, Y. et al. Extraordinarily strong interlayer interaction in 2D layered PtS2. Advanced Materials **28,** 2399–2407 (2016).
12. Yan, M. et al. High quality atomically thin PtSe2 films grown by molecular beam epitaxy. 2D Materials **4,** 045015 (2017).

13. Gupta, U. et al. Characterization of few-layer 1T-MoSe2 and its superior performance in the visible-light induced hydrogen evolution reaction. APL Materials **2,** 092802 (2014).

14. Song, Q. et al. Physical origin of Davydov splitting and resonant Raman spectroscopy of Davydov components in multilayer MoTe2. Physical Review B **93,** 115409 (2016).

16. Lui, C. H. et al. Observation of interlayer phonon modes in van der Waals heterostructures. Physical Review B **91,** 165403 (2015).

17. He, R. et al. Interlayer breathing and shear modes in NbSe2 atomic layers. 2D Materials **3,** 031008 (2016).

18. Chen, X. et al. Direct observation of interlayer coherent acoustic phonon dynamics in bilayer and few-layer PtSe2. Photonics Research **7,** 1416–1424 (2019).

19. Dong, S. et al. Ultralow-frequency collective compression mode and strong interlayer coupling in multilayer black phosphorus. Physical Review Letters **116,** 087401 (2016).

20. Tan, P. et al. The shear mode of multilayer graphene. Nature materials **11,** 294–300 (2012).

21. Li, X. et al. Rhombohedral-stacked bilayer transition metal dichalcogenides for high-performance atomically thin CMOS devices. Science Advances **9,** eade5706 (2023).

23. Kim, H. H. et al. Evolution of interlayer and intralayer magnetism in three atomically thin chromium trihalides. Proceedings of the National Academy of Sciences **116,** 11131–11136 (2019).

24. Telford, E. J. et al. Layered antiferromagnetism induces large negative magnetoresistance in the van der Waals semiconductor CrSBr. Advanced Materials **32,** 2003240 (2020).

25. McGuire, M. A. Crystal and magnetic structures in layered, transition metal dihalides and trihalides. Crystals **7,** 121 (2017).

26. Klein, D. R. et al. Enhancement of interlayer exchange in an ultrathin two-dimensional magnet. Nature Physics **15,** 1255–1260 (2019).

27. He, J., Hummer, K. & Franchini, C. Stacking effects on the electronic and optical properties of bilayer transition metal dichalcogenides MoS_2, $MoSe_2$, WS_2, and WSe_2. Phys. Rev. B **89,** 075409 (7 Feb. 2014).

28. Wei, S., Li, J., Liao, X. & Jin Hao amd Wei, Y. Investigation of Stacking Effects of Bilayer MoSSe on Photocatalytic Water Splitting. J. Phys. Chem. C **123,** 22570–22577 (36 2019).

29. Zhou, W., Chen, J., Yang, Z., Liu, J. & Ouyang, F. Geometry and electronic structure of monolayer, bilayer, and multilayer Janus WSSe. Phys. Rev. B **99,** 075160 (7 Feb. 2019).

30. Klein, D. R. et al. Enhancement of interlayer exchange in an ultrathin two-dimensional magnet. Nature Physics **15,** 1255–1260 (12 2019).

31. Bacaksiz, C. et al. Bilayer SnS_2: Tunable stacking sequence by charging and loading pressure. Phys. Rev. B **93,** 125403 (12 Mar. 2016).

32. Shu, H., Li, Y., Niu, X. & Wang, J. The stacking dependent electronic structure and optical properties of bilayer black phosphorus. Phys. Chem. Chem. Phys. **18,** 6085–6091 (8 2016).

33. Constantinescu, G., Kuc, A. & Heine, T. Stacking in Bulk and Bilayer Hexagonal Boron Nitride. Phys. Rev. Lett. **111,** 036104 (3 July 2013).

34. Marom, N. et al. Stacking and Registry Effects in Layered Materials: The Case of Hexagonal Boron Nitride. Phys. Rev. Lett. **105,** 046801 (4 July 2010).

35. Fang, L., Liang, W., Feng, Q. & Luo, S.-N. Structural engineering of bilayer PtSe2 thin films: a first-principles study. Journal of Physics: Condensed Matter **31,** 455001 (Aug. 2019).

36. Sivadas, N., Okamoto, S., Xu, X., Fennie, C. J. & Xiao, D. Stacking-Dependent Magnetism in Bilayer CrI3. Nano Letters **18.** PMID: 30408960, 7658–7664 (2018).

37. Gibertini, M. Magnetism and stability of all primitive stacking patterns in bilayer chromium trihalides. Journal of Physics D: Applied Physics **54,** 064002 (Nov. 2020).

38. Wilson, N. P. et al. Interlayer electronic coupling on demand in a 2D magnetic semiconductor. Nature Materials **20,** 1657–1662 (12 2021).

39. Li, A. et al. Coupling Stacking Orders with Interlayer Magnetism in Bilayer H-VSe2. Chinese Physics Letters **37,** 107101 (2020).

40. Zhu, Y. et al. Giant tunneling magnetoresistance in van der Waals magnetic tunnel junctions formed by interlayer antiferromagnetic bilayer $CoBr_2$. Phys. Rev. B **103,** 134437 (13 2021).

41. Lu, M., Yao, Q., Xiao, C., Huang, C. & Kan, E. Mechanical, Electronic, and Magnetic Properties of NiX2 (X = Cl, Br, I) Layers. ACS Omega **4,** 5714–5721 (2019).

42. Wang, H. & Qian, X. Electrically and magnetically switchable nonlinear photocurrent in PT-symmetric magnetic topological quantum materials. npj Computational Materials **6,** 199 (1 2020).
