# Supplementary material for: High-throughput computational stacking reveals emergent properties in natural van der Waals bilayers
Source: Nat Commun. 2024 Jan 31;15:932. doi: 10.1038/s41467-024-45003-w (PMC10831070; doi:10.1038/s41467-024-45003-w)
Supplement: Supplementary file 2 — Peer Review File [file 41467_2024_45003_MOESM2_ESM.pdf]

nature portfolio

Peer Review File

High-throughput computational stacking reveals emergent properties in natural van der Waals bilayers

Open Access This file is licensed under a Creative Commons Attribution 4.0 International License, which permits use, sharing, adaptation, distribution and reproduction in any medium or format, as long as you give appropriate credit to the original author(s) and the source, provide a link to the Creative Commons license, and indicate if changes were made. In the cases where the authors are anonymous, such as is the case for the reports of anonymous peer reviewers, author attribution should be to 'Anonymous Referee' followed by a clear attribution to the source work. The images or other third party material in this file are included in the article's Creative Commons license, unless indicated otherwise in a credit line to the material. If material is not included in the article's Creative Commons license and your intended use is not permitted by statutory regulation or exceeds the permitted use, you will need to obtain permission directly from the copyright holder. To view a copy of this license, visit http://creativecommons.org/licenses/by/4.0/.

Editorial Note: This manuscript has been previously reviewed at another journal that is not operating a transparent peer review scheme. This document only contains reviewer comments and rebuttal letters for versions considered at *Nature Communications*.

REVIEWERS' COMMENTS

Reviewer #2 (Remarks to the Author):

I am fully satisfied with the response of the Authors to my comments. The manuscript has considerably improved, and I recommend it for publication in its present form.

Reviewer #3 (Remarks to the Author):

I sincerely appreciate the effort made by the authors to revise the manuscript according to the suggestions by all reviewers. As already mentioned in my previous report, this manuscript is well suited for a journal like Nature Communications and, with these final revisions, I would consider it ready for publication in this journal.
